# 5′-NucA-SMCC-DM1 and 5′-NucA-SPDMV-DM1 are Potent Aptamer–Drug Conjugates against Pancreatic Cancer

**DOI:** 10.1021/acsomega.5c11364

**Published:** 2026-02-03

**Authors:** Hong Dai, Razack Abdullah, Wenqiong Huang, Xiaoli Chen, Aiping Lu, Kenneth Cp Cheung

**Affiliations:** † Department of Chemistry, 428699 The Hong Kong University of Science and Technology, Clear Water, Bay, Kowloon, Hong Kong SAR 999077, China; ‡ Law Sau Fai Institute for Advancing Translational Medicine in Bone and Joint Diseases, School of Chinese Medicine, 26679 Hong Kong Baptist University, Kowloon, Hong Kong SAR 999077, China; § Phenome Research Center, School of Chinese Medicine, 71046 Hong Kong Baptist University, Kowloon, Hong Kong SAR 999077, China

## Abstract

Pancreatic cancer is one of the most lethal cancers, characterized by low survival rates due to a complex tumor microenvironment, late-stage diagnosis and, notably, the limited effectiveness of current treatments. First-line therapies, such as gemcitabine and nab-paclitaxel, often lead to unexpected side effects. Mertansine, which is a more potent cytotoxic agent, faces similar challenges. In response, we designed and synthesized a highly water-soluble conjugate of an antinucleolin aptamer (NucA) and mertansine (NucA-DM1) to enhance the delivery of DM1 specifically to pancreatic tumor cells. Our *in vitro* studies demonstrated that the cytotoxic activity of this conjugate could retain potency compared to DM1 alone, with significant accumulation observed in pancreatic tumor cells rather than in normal cell lines. Additionally, 5′-NucA-SMCC-DM1 and 5′-NucA-SPDMV-DM1 conjugates exhibited excellent stability in serum. Notably, 3′-Cy5-5′-NucA-SMCC-DM1 was primarily taken up by PANC-1 cells through macropinocytosis. Further investigations into the antitumor activity and cell cycle dynamics indicated that the conjugation of NucA and DM1 minimally impacted the 5′-linked aptamer–drug conjugate (ApDC), whereas the 3′-linked ApDC remained unaffected. Our findings also confirmed that SMCC- and SPDMV-linked ApDCs retained stability in human serum for up to 48 h. Flow cytometry and confocal microscopy analyses further illustrated the excellent targeting capabilities of these conjugates in pancreatic cancer cell lines PANC-1 and MIA PaCa-2, in contrast to normal cells such as MIHA (normal human liver cells). Two candidates, 5′-NucA-SMCC-DM1 and 5′-NucA-SPDMV-DM1, were selected based on *in vitro* evaluations and exhibited potent antitumor efficacy with significantly decreased toxicity to the liver and heart compared with DM1 alone in xenografted mice.

## Graphical abstract

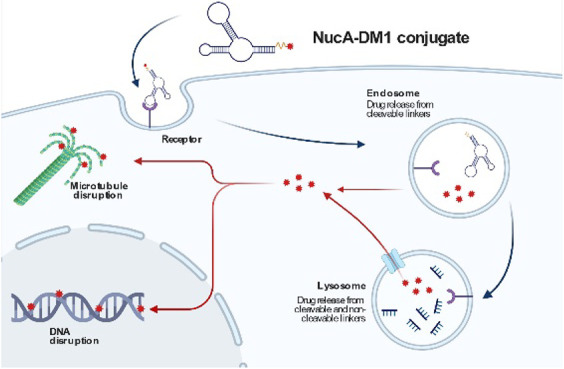

## Introduction

Pancreatic cancer is one of the most fatal cancers, with a 5-year overall survival rate of approximately 10%, and it is becoming an increasingly common cause of cancer mortality.^1^ The low survival rate can mainly be attributed to the delayed detection of patients, who often show no specific symptoms at the early stage, thereby leading to advanced stages when symptoms become obvious.^2,3^ The tumor microenvironment of pancreatic cancer also consists of complicated genetic, epigenetic, and metabolic alterations, as well as equally complex interactions between cancer cells and stromal cells, immune cells, and endothelial cells, forming a dense mechanical barrier surrounding pancreatic tumors, which also contributes to the treatment challenge of fibrosis.^4,5^ The standard treatments for pancreatic cancer largely depend on the chemotherapy typically including gemcitabine- and paclitaxel-based combinations.^6^ Despite chemotherapy providing clinical benefits, it is still unsatisfactory in achieving long-term survival. One strategy to improve overall treatment efficacy is the introduction of antibody–drug conjugates (ADCs) which consist of a targeting moiety and drugs connected by various types of linkers.^7^ Since the first approval of Gemtuzumab ozogamicin in 2000 for the treatment of CD33-positive acute myeloid leukemia, there have been 13 ADCs approved by the FDA to date. However, no ADCs have been approved by the FDA for the treatment of pancreatic cancer to date, despite the overall treatment efficacy progress made in other cancers (e.g., HER2-positive breast cancer, acute myeloid leukemia) by elevating drug accumulation in tumors with reduced systemic cytotoxicity.^8^ Maytansine (MTS), a family of cytotoxins with a macrolide structure, is an exceptionally potent anticancer agent, which shows excellent cytotoxicity to a range of cancer cell lines, including pancreatic cancer.^9^ However, its inability to distinguish cancer cells from normal cells can induce severe systemic toxicity, which restrains its further application in clinical treatment. Currently, antibody–drug conjugates of MTS derivatives, such as mertansine (DM1), have been developed to increase tumor targeting and reduce toxicity, and many antibody–drug derivative conjugates have entered clinical trials, such as cantuzumab ravtansine (HuC242-DM4; IMGN242) for the treatment of solid tumors, including pancreatic cancer. Trastuzumab emtansine (brand name: Kadcyla) has also been approved by the FDA for the treatment of HER2-positive unresectable or metastatic breast cancer.^10,11^

Despite their advantages, antibody–drug conjugates (ADCs) face several drawbacks, including immunogenicity and poor tissue penetration. In contrast, nucleic acid aptamers, which are short single-stranded nucleic acids that can bind specific targets in a manner similar to antibodies, exhibit limited immunogenicity, own a smaller size favorable for superior tissue penetration, and have a lower likelihood of developing resistance, making them valuable tools for tumor targeting. For instance, the antinucleolin aptamer NucA (AS1411) has demonstrated effective tumor targeting with a favorable safety profile in clinical settings. NucA binds to nucleolin, a protein normally expressed in the nucleus and cytosol but overexpressed both intracellularly and on the cell surface, especially in many types of cancers, including pancreatic cancer.^12–14^ High levels of cell surface nucleolin are linked to increased cancer malignancy, metastasis, and a poorer prognosis for patients,^15^ making it a potential diagnostic or therapeutic target. Despite the fact that conjugations between aptamers and cytotoxic payloads have been reported,^16–19^ even with DM1 ^20–22^ against many indications, the conjugation between NucA and DM1 against pancreatic cancer still remains unexplored.

To leverage the benefits of NucA, we aimed to conjugate it with commercially available DM1 to create an aptamer–drug conjugate (ApDC). This approach seeks to enhance targeting capabilities while reducing toxicity. We selected the noncleavable linker SMCC, inspired by the FDA-approved ADC drug Kadcyla. Additionally, we incorporated cleavable linkers such as SPDP, SPP, and SPDMV to potentially enhance bystander killing of neighboring nontargeted tumor cells through the release of diffusible cytotoxic metabolites.

The antitumor activity and cell cycle assays indicated that the modification of NucA to DM1 had minimal impact on the 5′-linked ApDC, while it significantly affected the 3′-linked ApDC. We also found that SMCC- and SPDMV-linked ApDCs maintained stability in human serum for up to 48 h. Further analyses using flow cytometry and confocal microscopy demonstrated the excellent targeting capabilities of these conjugates in pancreatic cancer cell lines PANC-1 and MIA PaCa-2, compared to normal cells such as MIHA. The *in vivo* antitumor efficacy also demonstrated the potency of the combination with reduced cytotoxicity compared with DM1.

## Results

### Chemical Synthesis of NucA-DM1 Conjugates and Derivatives

To link NucA with DM1, NH_2_-AS1411 (3′- or 5′-linked) was directly conjugated with four types of linkers containing activated carboxylic acid, followed by the conjugation between the thiol group of DM1 and N-maleimide. Taking the 3′-linked and 5′-linked differences into consideration, 16 conjugates were obtained, including 5′-NucA-SMCC-DM1, 5′-NucA-SPP-DM1, 5′-NucA-SPDP-DM1, and 5′-NucA-SPDMV-DM1, as well as 3′-NucA-SMCC-DM1, 3′-NucA-SPP-DM1, 3′-NucA-SPDP-DM1, and 3′-NucA-SPDMV-DM1. To further investigate the cellular internalization and *in vivo* biodistribution, we synthesized two types of conjugates, including 3′-Cy5-5′-NucA-SMCC-NucA and 3′-Cy5-5′-NucA-SPDMV-NucA. All the final conjugates were purified by HPLC, desalted, and confirmed by HR-MS (Figure 1 and Supporting Information, Figure S10–S33).

**Figure 1:**
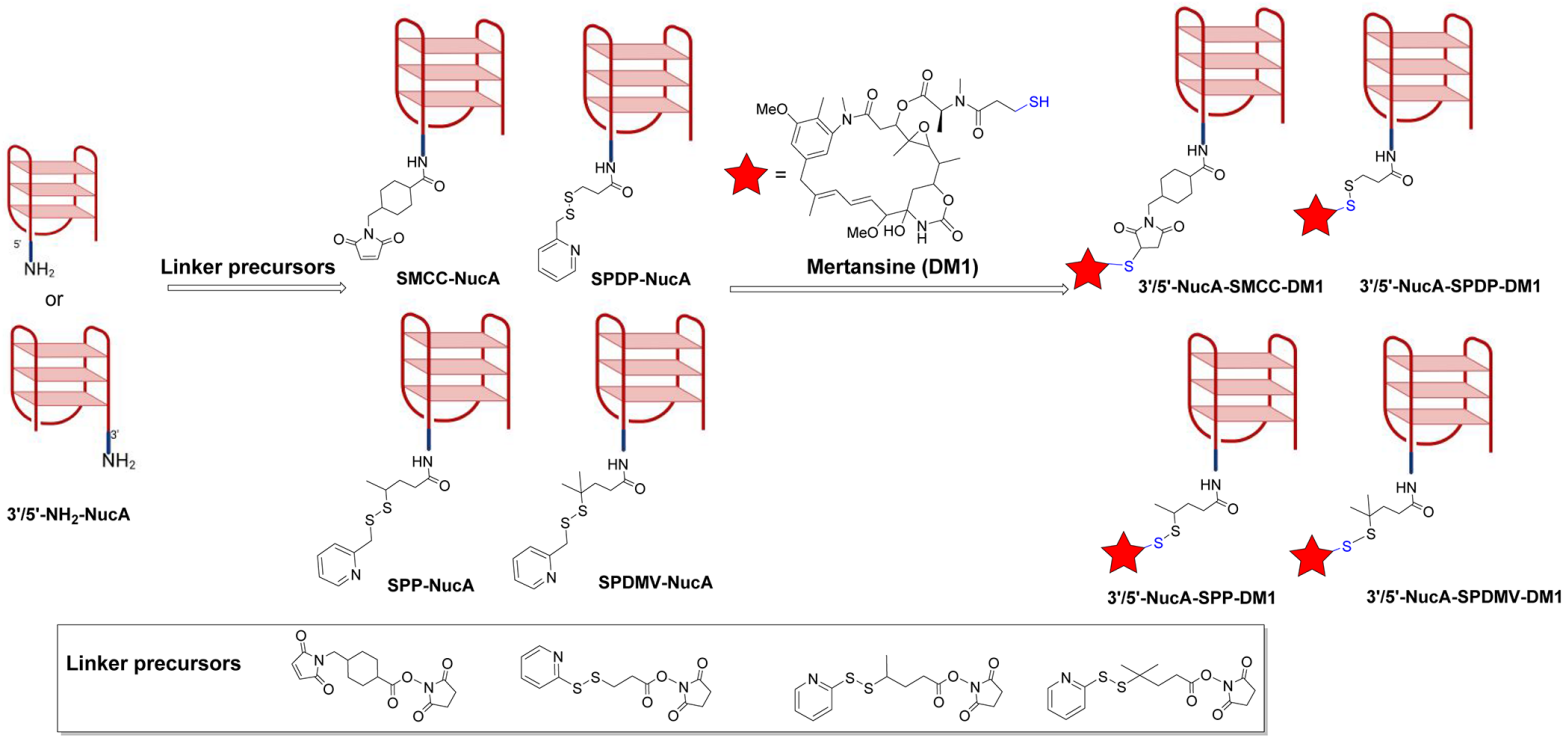
Schematic diagram of chemical synthesis of NucA-linker-DM1 conjugates. 3′/5′-NH_2_–NucA (or 3′-Cy5 −5′-NH_2_–NucA) was coupled with excessive linker precursors through activated carboxyl groups, followed by conjugation to DM1 containing a reactive thiol group. The obtained conjugates were further purified, desalted, characterized, and quantified prior to any evaluations.

### Antiproliferative Activity of Synthesized ApDCs

To investigate whether the modification of NucA could affect the antitumor activity of conjugated DM1, all the synthesized ApDCs were tested for their *in vitro* antiproliferative activity against two pancreatic cancer cell lines, PANC-1 , and MIA PaCa-2, as well as one normal cell line, MIHA. The results indicated that all the 5′-linked ApDCs, including 5′-NucA-SMCC-DM1, 5′-NucA-SPP-DM1, 5′-NucA-SPDP-DM1, and 5′-NucA-SPDMV-DM1, exhibited slightly lower antiproliferative activity than DM1 (Figure 2). The EC_50_ values of the 5′-linked ApDCs in PANC-1 and MIA PaCa-2 were all below the 10 nM level (Table 1) which is of the same order of magnitude compared with DM1. However, most of the 3′-linked ApDCs, including 3′-NucA-SMCC-DM1, 3′-NucA-SPP-DM1, and 3′-NucA-SPDMV-DM1, showed a remarkably decreased antitumor activity against PANC-1 and MIA PaCa-2 compared to DM1, and they were also inferior to the 5′-linked ApDCs. The most significant difference was observed between 5′-NucA-SMCC-DM1 and 3′-NucA-SMCC-DM1, with EC_50_ values of 23.14 and 179.6 nM in PANC-1, and 9.72, and 113.4 nM in MIA PaCa-2, respectively. In terms of cytotoxicity to MIHA cells, all of the 3′-linked or 5′-linked ApDCs showed decreased toxicity in comparison with DM1, as well as in PANC-1 and MIA PaCa-2 (Figure 2). In contrast, the control aptamer CRO-based conjugates showed slightly lower antiproliferative activity against both PANC-1 and MIA PaCa-2 cells but higher cytotoxicity against MIHA (Figure S1 and Table S1).

**Figure 2:**
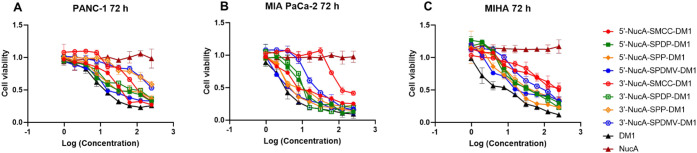
Antiproliferative activity of synthesized NucA-DM1 conjugates in PANC-1, MIA PaCa-2, and MIHA. The cell viability curves of NucA-conjugates against (A) PANC-1 cells, (B) MIA PaCa-2 cells, and (C) MIHA cells were revealed by the CCK-8 assay as indicated within 72 h of incubation at 37 °C. Data were presented as mean ± SD of three independent experiments, and each was measured in triplicate (*n* = 3). S.D., standard deviation.

**Table 1:** EC_50_ Values of Synthesized ApDCs against PANC-1, MIA PaCa-2, and MIHA Cells

	**EC**_**50**_ **(nM, 72 h)**					
Entry	PANC-1		MIA PaCa-2		MIHA	
DM1	7.79 ± 0.84		2.50 ± 0.82		2.70 ± 0.64	
**Linker**	**5′-ApDC**	**3′-ApDC**	**5′-ApDC**	**3′-ApDC**	**5′-ApDC**	**3′-ApDC**
SMCC	23.14 ± 0.67	179.6 ± 1.08	9.72 ± 0.57	113.4 ± 0.60	44.03 ± 1.23	81.83 ± 2.16
SPDP	9.85 ± 0.66	15.91 ± 0.72	8.87 ± 0.84	8.83 ± 0.83	24.15 ± 1.00	27.34 ± 1.02
SPP	9.88 ± 0.60	45.97 ± 0.50	3.33 ± 0.95	13.27 ± 1.05	16.00 ± 0.99	32.74 ± 1.84
SPDMV	10.18 ± 0.61	56.83 ± 2.17	2.63 ± 0.95	16.56 ± 1.93	16.42 ± 0.92	63.57 ± 2.84

To further examine whether the NucA-DM1 conjugates could exert the same antitumor mechanism as DM1, 5′-NucA-SMCC-DM1 was selected as one representative to be subjected to the cell cycle assay. The untreated PANC-1 or MIA PaCa-2 cells showed a significant fraction of about 50% in the G0/1 cell cycle. However, 5′-NucA-SMCC-DM1-treated PANC-1 or MIA PaCa-2 cells showed almost no G0/1 fraction, and the fraction of cells in the G2/M and S phases was remarkably increased (Figure 3), showing a similar trend compared with simple DM1. Based on the above results, the 5′-linked ApDcs were selected as the next screening candidates.

**Figure 3:**
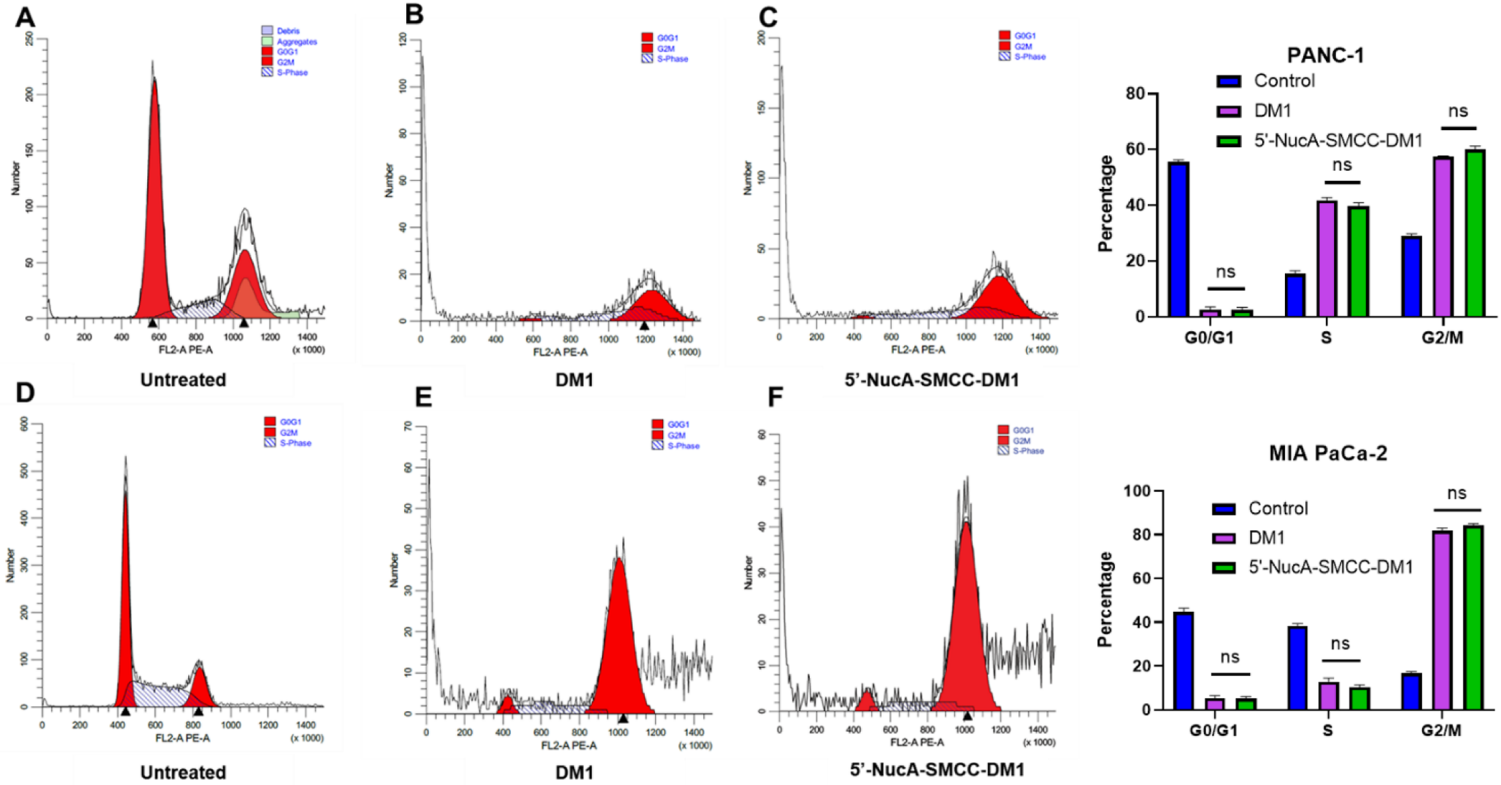
Cell cycle analysis was conducted through propidium iodide staining. The flow cytometry for the PANC-1 cells treated with (A) blank control, (B) DM1, and (C) 5′-NucA-SMCC-DM1, and the MIA PaCa-2 cells treated with (D) blank control, (E) DM1, and (F) 5′-NucA-SMCC-DM1 of different cell cycle phases, showed percentages of cell cycle phases on the right. Data were presented as mean ± SD of three independent experiments, and each was measured in triplicate (*n* = 3). One-way ANOVA was used for statistical analysis, and the significance levels were indicated as *p < 0.05, **p < 0.01, ***p < 0.001, ****p < 0.0001. ns, no significance. S.D., standard deviation.

### Serum Stability and Release in PANC-1 Cells

5′-NucA-SMCC-DM1, 5′-NucA-SPDP-DM1, 5′-NucA-SPP-DM1, and 5′-NucA-SPDMV-DM1 were further selected for the serum stability assay, considering that these 5′-linked ApDCs showed significantly higher antitumor activity than 3′-linked ApDCs. The results showed that all of the above ApDCs could remain stable within 24 h, whereas 5′-NucA-SPDP-DM1 and 5′-NucA-SPP-DM1 showed significant degradation within 48 h. In this way, 5′-NucA-SMCC-DM1 and 5′-NucA-SPDMV-DM1 showed the best stability within 48 h in 80% human serum (Figure 4). Compared to NucA, the binding affinity of 5′-NucA-SMCC-DM1 and 5′-NucA-SPDMV-DM1 to nucleolin slightly increased to 163 ± 36 and 148 ± 17 nM, respectively (Figure S2).

**Figure 4:**
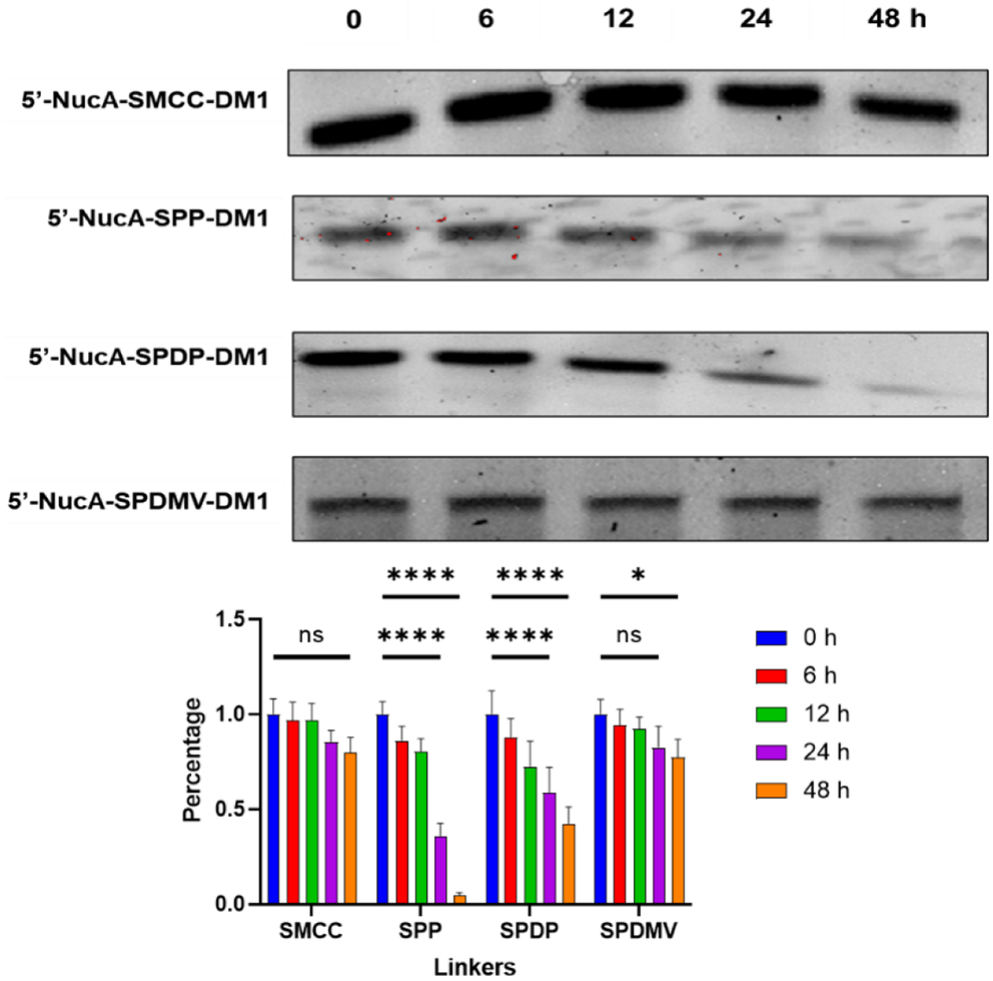
Stability of synthesized conjugates in human normal serum. Four conjugates, including 5′-NucA-SMCC-DM1, 5′-NucA-SPP-DM1, 5′-NucA-SPDP-DM1, and 5′-NucA-SPDMV-DM1, were coincubated with human normal serum (80%) at 37 °C at designated time slots of 0, 6, 12, 24, and 48 h, followed by polyacrylamide gel electrophoresis (PAGE) and nucleic acid visualization. The values represented the remaining percentage of integrated density compared to the control (0 h) after background subtraction. Data were presented as mean ± SD of three independent experiments, and each was measured in triplicate (*n* = 3). One-way ANOVA was used for statistical analysis, and the significance levels were indicated as *p < 0.05, **p < 0.01, ***p < 0.001, ****p < 0.0001. ns, no significance. S.D., standard deviation.

Furthermore, the DM1 release from 5′-NucA-SMCC-DM1, 5′-NucA-SPDP-DM1, 5′-NucA-SPP-DM1, and 5′-NucA-SPDMV-DM1 was tested after coincubation with PANC-1 cells (Figure S3). The results showed that all the conjugates were fully degraded within 24 hours of incubation. SPDP- and SPP-linked conjugates degraded much faster than SPDMV- and SMCC-linked conjugates. To further investigate the in vitro bystander killing effect, we tested the cytotoxicity of 5′-NucA-SMCC-DM1 and 5′-NucA-SPDMV-DM1 in cocultured PANC-1 and MIHA cells. The results indicated that 5′-NucA-SPDMV-DM1 exerted almost the same killing effect (10.18 versus 10.52), while the EC_50_ of 5′-NucA-SMCC-DM1 slightly increased to 30.31 nM compared to the original 23.14 nM (Figure S4 and Table S2).

### Cellular-Bound Conjugates and Internalization

To investigate whether the NucA modification enhances cellular binding and internalization in PANC-1 and MIA PaCa-2 cells, we conducted flow cytometry analyses. 3′-Cy5-NucA was conjugated to DM1 using SMCC and SPDMV linkers, resulting in the constructs 3′-Cy5-5′-NucA-SMCC-DM1 and 3′-Cy5-5′-NucA-SPDMV-DM1, respectively. Both conjugates demonstrated dose-dependent responses (Figure 5), with 3′-Cy5-5′-NucA-SMCC-DM1 exhibiting higher signals in PANC-1 and MIA PaCa-2 cells compared to 3′-Cy5-5′-NucA-SPDMV-DM1. Notably, both labeled ApDCs showed significantly lower signals in MIHA cells (Figure 6), indicating selective cellular binding and internalization of NucA-conjugated DM1 by cancer cells. Notably, DNase treatment to remove surface-bound conjugates did not show a significant difference compared to the untreated group (Figure S5).

**Figure 5:**
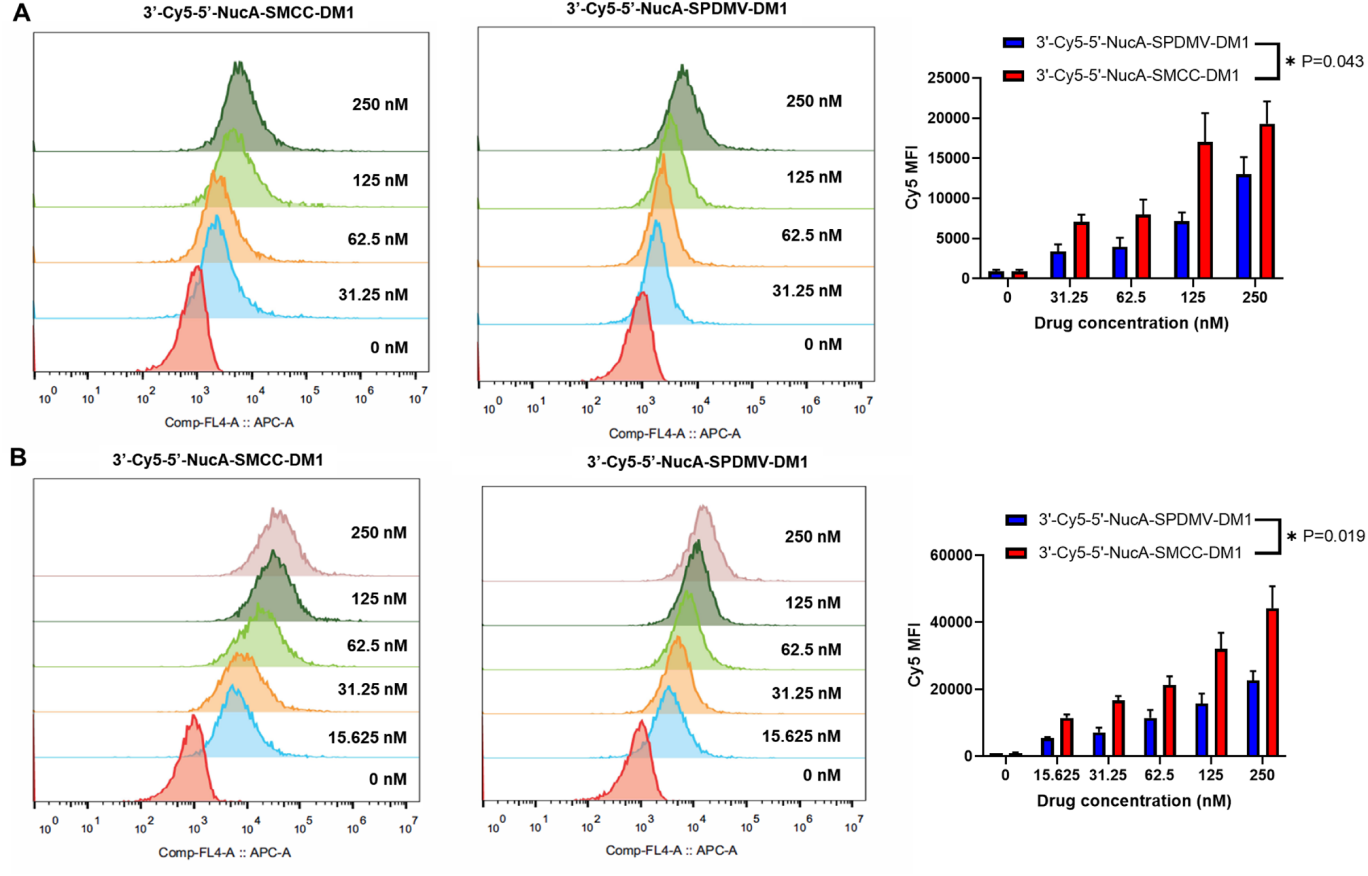
Effect of conjugated DM1 on cellular binding and internalization analysis by flow cytometry. Cy5 MFI of 3′-Cy5-5′-NucA-SMCC-DM1 and 3′-Cy5-5′-NucA-SPDMV-DM1 in (A) PANC-1 cells and (B) MIA PaCa-2 cells within 2 h of incubation at 37 °C. Untreated control was used for 3′-Cy5-5′-NucA-SMCC-DM1 and 3′-Cy5-5′-NucA-SPDMV-DM1 in PANC-1, and the vertical stretching can offer better visualization for each concentration. Data were presented as mean ± SD of three independent experiments (*n* = 3), and each was measured in triplicate. Student’s *t*-test was used for statistical analysis (250 nM concentration), and the significance levels were indicated as *p < 0.05, **p < 0.01, ***p < 0.001, ****p < 0.0001. ns, no significance. MFI, mean fluorescence intensity. S.D., standard deviation. APC-A, allophycocyanin by area.

**Figure 6:**
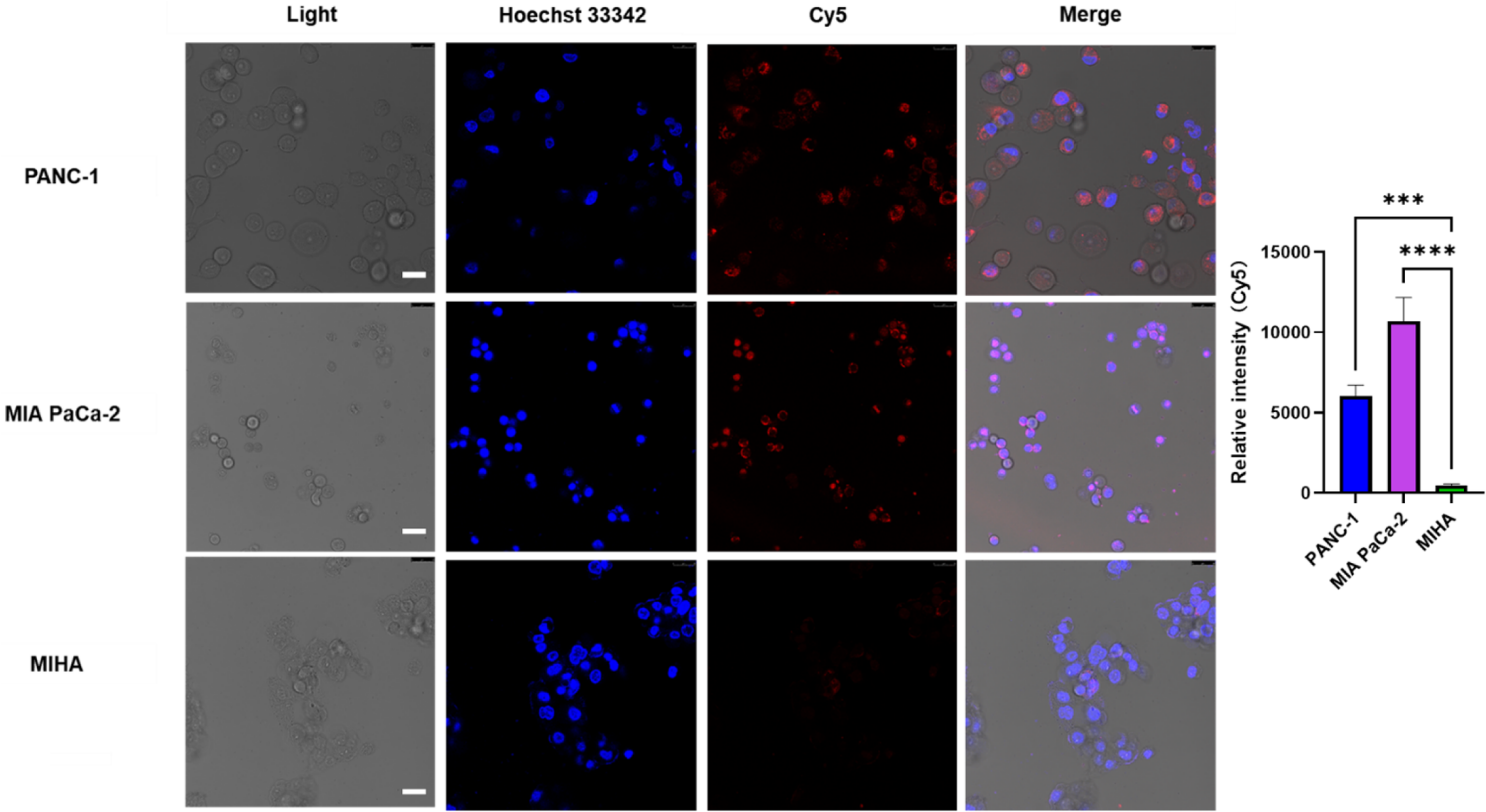
Effect of conjugated DM1 on cellular binding and internalization by confocal microscopy. 250 nM 3′-Cy5-5′-NucA-SMCC-DM1 (red) was incubated with PANC-1, MIA PaCa-2, and MIHA cells, respectively, at 37 °C for 2 h. The nuclei were counterstained with Hoechst 33,342 (blue). Scale bar, 25 μm (the upper right black bar is the original bar, and the white bar is for better visualization). Error bars indicate mean ± SD (*n* = 5 per group). Each replicate is from one biological experiment, quantified with 10 independent fields of view. One-way ANOVA was used for statistical analysis, and the significance levels were indicated as *p < 0.05, **p < 0.01, ***p < 0.001, ****p < 0.0001. ns, no significance. S.D., standard deviation.

Additionally, we examined the cellular binding and internalization of 250 nM 3′-Cy5-5′-NucA-SMCC-DM1 and 3′-Cy5-5′-CRO-SMCC-DM1 in PANC-1, MIA PaCa-2, and MIHA cells using confocal microscopy. This analysis revealed a consistent trend of increased cellular binding and internalization in the pancreatic cancer cells compared with the normal MIHA cells for 3′-Cy5-5′-NucA-SMCC-DM1. In contrast, 3′-Cy5-5′-CRO-SMCC-DM1 showed significantly lower responses in both PANC-1 and MIA PaCa-2 cells (Figure S6). To further explore the mechanism of internalization, we incubated 250 nM 3′-Cy5-5′-NucA-SMCC-DM1 and 3′-Cy5-5′-CRO-SMCC-DM1 with Alexa Fluor 488-labeled endocytic markers, followed by confocal microscopy analysis. The results indicated a significantly higher colocalization of 3′-Cy5-5′-NucA-SMCC-DM1 with dextran (a marker for macropinocytosis) compared to transferrin (a marker for clathrin-mediated endocytosis) or cholera toxin (a marker for caveolae-mediated endocytosis) (Figure 7). However, no significant colocalization signals of 3′-Cy5-5′-CRO-SMCC-DM1 could be observed (Figure S7), demonstrating a possible involvement of macropinocytosis in ApDC cellular internalization. The reduced binding and internalization of 3′-Cy5-5′-NucA-SMCC-DM1 when cells were pretreated with EIPA (an inhibitor of macropinocytosis) at various concentrations could also be observed.

**Figure 7:**
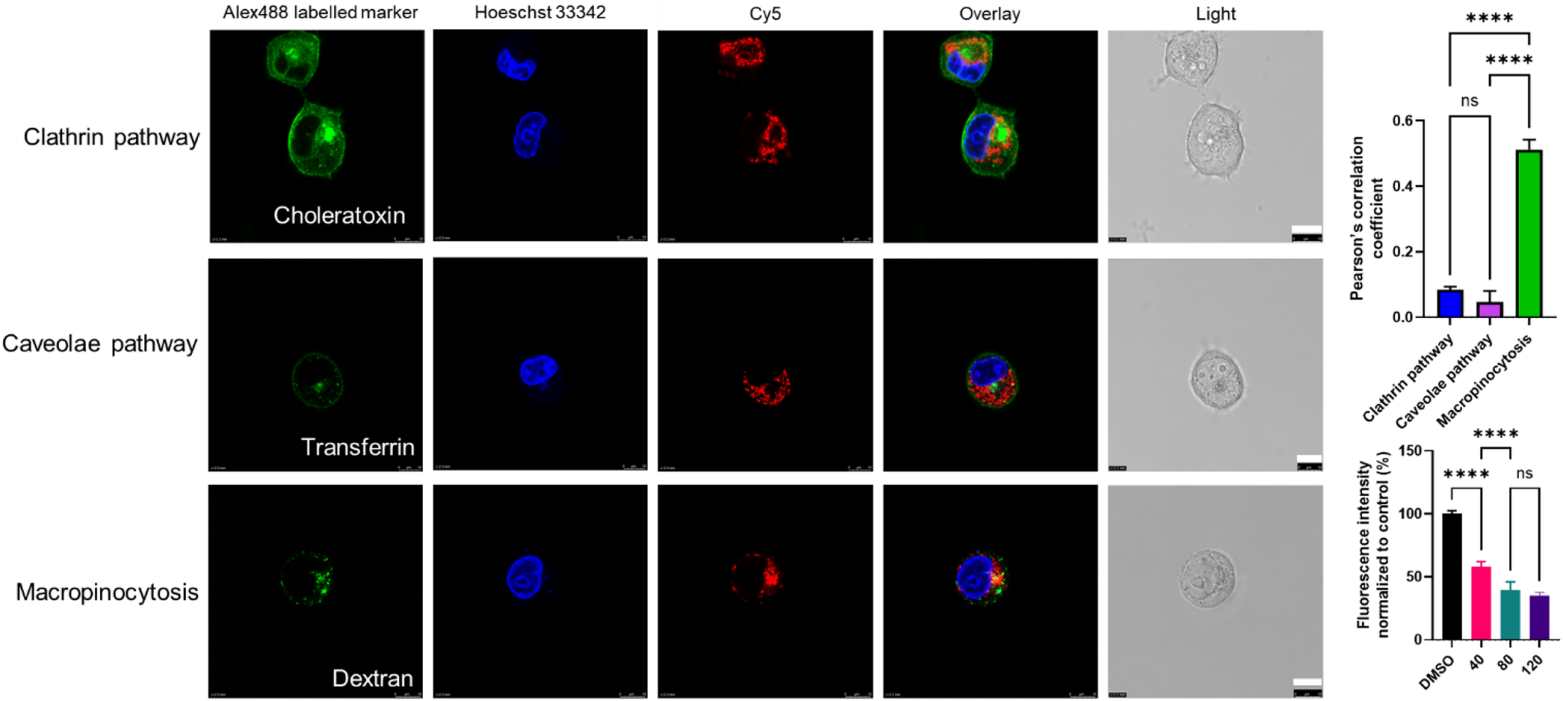
Effect of conjugated DM1 on cellular binding and internalization. 250 nM 3′-Cy5-5′-NucA-SMCC-DM1 was incubated with three Alexa Fluor 488-labeled endocytic markers (transferrin, cholera toxin, and dextran; green) in PANC-1 cells, and the nuclei were counterstained with Hoechst 33,342 (blue). Scale bar, 10 μm (the lower right black bar is the original bar; the white bar is for better visualization). Error bars indicate mean ± SD (*n* = 5 per group). Each replicate is from one biological experiment, quantified with 10 independent fields of view. One-way ANOVA was used for statistical analysis, and the significance levels were indicated as *p < 0.05, **p < 0.01, ***p < 0.001, ****p < 0.0001. ns, no significance. S.D., standard deviation.

### Antitumor Efficacy *In Vivo*

To assess whether the NucA modification improved the biodistribution of DM1, we tested the fluorescence signal intensity after subcutaneous injection with 3′-Cy5-5′-NucA-SMCC-DM1, 3′-Cy5-5′-NucA-SPDMV-DM1, 3′-Cy5-5′-CRO-SMCC-DM1, and 3′-Cy5-5′-CRO-SPDMV-DM1. It was shown that the Cy5 fluorescence intensities in tumor tissues collected from the 5′-NucA-SMCC-DM1- and 5′-NucA-SPDMV-DM1-treated mice were significantly higher than those from the 5′-CRO-SMCC-DM1- and 5′-CRO-SPDMV-DM1-treated mice at 4 h after intravenous injection, respectively (Figure S8). The overall signals in livers and kidneys from the 5′-NucA-SMCC-DM1 and 5′-NucA-SPDMV-DM1 conjugates were significantly lower than those of the related CRO conjugates. The serum half-life of 5′-NucA-SMCC-DM1 and 5′-NucA-SPDMV-DM1 was evaluated to be 4.37 and 3.58 h, respectively (Figure S9).

To assess whether the NucA modification improved the efficacy of DM1, we established several treatment groups, including the treatment groups: 5′-NucA-SPDMV-DM1 and 5′-NucA-SMCC-DM1. The control groups consisted of PBS, NucA, and DM1, 5′-CRO-SPDMV-DM1, and 5′-CRO-SMCC-DM1. A dosage of conjugate equivalent to 0.1 mg DM1/kg was administered via IV injection to three groups of mice twice a week for 4 weeks. After the treatment, the mice were sacrificed, and their pancreatic tumors were harvested for size measurement (Figure 8A). As anticipated, the negative control groups, PBS and NucA, showed no statistically significant differences. 5′-CRO-SPDMV-DM1 and 5′-CRO-SMCC-DM1 groups also showed a similar trend compared to the simple DM1 group. In contrast, 5′-NucA-SPDMV-DM1 and 5′-NucA-SMCC-DM1 resulted in significant tumor shrinkage compared to DM1, 5′-CRO-SPDMV-DM1, and 5′-CRO-SMCC-DM1. Notably, tumors treated with 5′-NucA-SPDMV-DM1 and 5′-NucA-SMCC-DM1 demonstrated a statistically significant reduction in size compared with DM1 and the respective CRO control groups.

**Figure 8:**
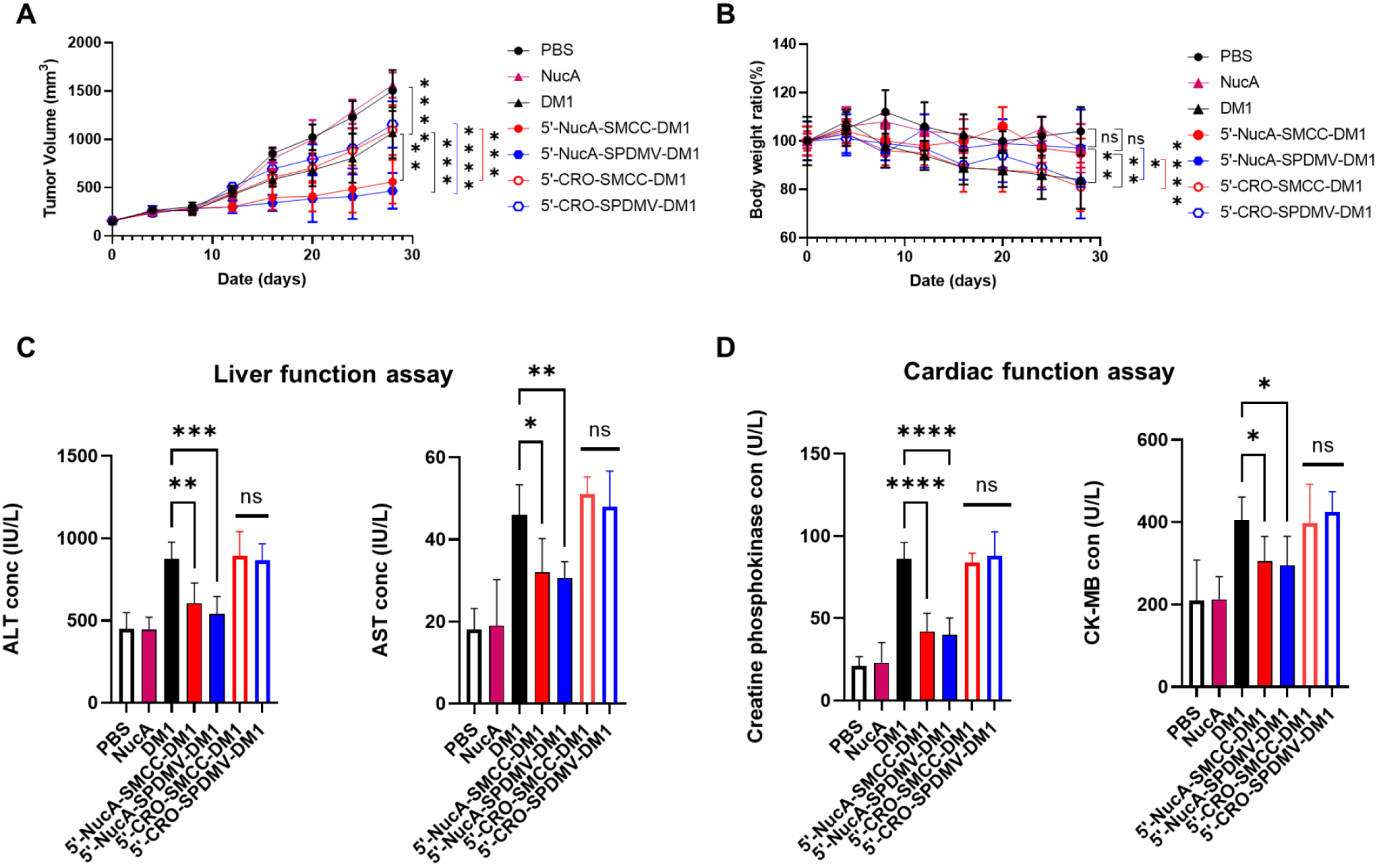
*In vivo* antitumor efficacy and safety evaluations for 5′-NucA-SPDMV-DM1 and 5′-NucA-SMCC-DM1. (A) Analysis of tumor volume after 4 weeks of various treatments administered via intravenous injection. (B) Body weight changes after 4 weeks of various treatments indicated in xenografted mice. (C) The ELISA results for two liver function enzymes, AST and ALT, as well as (D) cardiac function enzymes, CK-MB and CPK, were analyzed in pancreatic tumors treated with two primary comparison groups: 5′-NucA-SPDMV-DM1/5′-NucA-SMCC-DM1 and DM1. Additionally, control treatment groups were included: PBS, NucA. Data were mean ± SD of each grouped mice (*n* = 6 per group). Two-way ANOVA (for A and B, multiple comparisons) and one-way ANOVA (for C and D) were used for statistical analysis, and the significance levels were indicated as *p < 0.05, **p < 0.01, ***p < 0.001, ****p < 0.0001. ns, no significance. The listed comparison groups are DM1 vs 5′-NucA-SPDMV-DM1/5′-NucA-SMCC-DM1, 5′-NucA-SPDMV-DM1 vs 5′-CRO-SPDMV-DM1 (blue), and 5′-NucA-SMCC-DM1 vs 5′-CRO-SMCC-DM1 (red). S.D., standard deviation.

### NucA Modification Could Reduce Side Effects on Major Organs

Since DM1 in ADC has severe side effects, typically hepatotoxicity^23^ and cardiotoxicity,^24^ we mainly assessed body weight (Figure 8B) as well as liver and cardiac function through Enzyme-Linked Immunosorbent Assay (ELISA) kits after the treatment with 5′-NucA-SPDMV-DM1 and 5′-NucA-SMCC-DM1, using PBS, NucA, and DM1 as controls. Specifically, liver function was assessed based on two key liver enzymes: aspartate aminotransferase (AST) and alanine aminotransferase (ALT), respectively. Cardiac function was evaluated by measuring creatine phosphokinase (CPK) with a Creatine Kinase Activity Assay Kit and creatine kinase myocardial bound protein (CK-MB) using a Mouse CK-MB ELISA Kit. Crucially, the increases in AST, ALT, CPK, and CK-MB levels were significantly inhibited by 5′-NucA-SPDMV-DM1 and 5′-NucA-SMCC-DM1 treatment compared to DM1 treatment (Figure 8C, D).

## Discussion

Mertansine is a potent antineoplastic drug and has been used as the payload in the FDA-approved ADC drug Kadcyla. However, mertansine can accumulate in both normal tissue and tumors, thus leading to severe side effects. The conjugation strategy has always been a good choice for addressing inherent defects.^25^ In this study, we armed DM1 with one nucleolin aptamer, NucA, to selectively deliver this drug to pancreatic cancer cells while retaining its potency within a therapeutic window. For the linker selection, we carefully considered two types of linkers, including both cleavable and noncleavable. Theoretically, the cleavable linker should work better than the noncleavable linker because it can release the active drug form instead of the drug with certain modifications. For example, the vast majority of ADCs in clinical development have adopted cleavable linkers.^26^ However, there are also some exceptions, such as Trastuzumab-DM1, where conjugation through the noncleavable linker SMCC offered improved efficacy, pharmacokinetics and reduced toxicity compared to other disulfide linkers.^10^ Among the cleavable linkers, acid-cleavable and reducible disulfides are already clinically established methods. However, acid-cleavable linkers have proved difficult to strictly discriminate between pH 5 and pH 7.4, which is required for the efficient release of the active drug. Protease-cleavable linkers adopted in ADCs can be recognized and cut by lysosomal proteases, thereby triggering drug release. Reducible disulfide linkers, which undergo cleavage by intracellular high-concentration glutathione triggers (1–10 mM) but remain stable in blood plasma due to low glutathione concentration (1–20 μM), are the most prominent class of chemically cleavable motifs. Additionally, with the addition of steric protection by α-methyl around the disulfide, the linker becomes less susceptible to reduction. Therefore, a noncleavable linker (SMCC) and disulfide-cleavable linkers, including SPDP (no α-methyl), SPP (one α-methyl), and SPDMV (two α-methyls), were introduced.

The antiproliferative activity indicated that all 5′-linked ApDCs could exert slightly lower antitumor activity, still with potency retention in the therapeutic window compared to DM1, whereas 3′-linked ApDCs showed a significant descending trend. It might be hypothesized that the conjugation to the 5′-position or 3′-position of NucA could make a difference in the binding affinity toward nucleolin, leading to the internalization difference of DM1. On the other hand, linker cleavage differences could also matter. If the linkers are easier to cleave (SPDP > SPP > SPDMV, while SMCC is noncleavable), the rapid release of the active drug can impose better activity. The overall antitumor activity likely depends on the superimposed effects of binding affinity and linker cleavage of the active drug. For the CRO-linked conjugates, almost all the synthesized CRO–DM1 conjugates exhibited slightly lower antiproliferative activity against PANC-1 cells and MIA PaCa-2 cells but higher cytotoxicity against MIHA cells, demonstrating the specific role of the conjugation of NucA other than CRO.

ApDCs serve as prodrugs in our design,^27,28^ where they remain stable in the circulation system, mainly accumulate at the desired antigen-expressing cells, and release the active drug form after endocytosis into cells. The poor pharmacokinetic performance of ApDCs is influenced by two major factors: aptamer degradation by nucleases and rapid renal filtration due to the small molecular size of aptamers. From the obtained results of polyacrylamide gel electrophoresis (PAGE), we can see that the more α-methyl groups ApDCs have, the more stability we can observe. A maximum of 48 h of stability could be observed for the SPDMV linker, which is our set ending point. For ADCs, the release of the active drug maytansinoid from trastuzumab-SMCC-DM1 was negligible over 7 days, but only 11% of trastuzumab-SPP-DM1 remained in the circulation after 7 days (the release of maytansinoid can reach more than 80%).^10^ In the future, certain modifications, such as the 2′ position of ribose with fluoro (F), amino (NH_2_), O-methyl (OCH_3_) and phosphorothioate backbone introduction, could be adopted to increase the serum stability and affinity of aptamers.^29^

The cellular binding and internalization of representative 5′-NucA-SMCC-DM1 and 5′-NucA-SPDMV-DM1 are much higher in PANC-1 and MIA PaCa-2 than in MIHA, which is likely due to the expression level difference of nucleolin (Figure S10).^14^ For the *in vivo* biodistribution, both 5′-NucA-SMCC-DM1 and 5′-NucA-SPDMV-DM1 achieved significantly higher accumulation in tumor sites compared to 5′-CRO-SMCC-DM1 and 5′-CRO-SPDMV-DM1, indicating the targeted delivery led by NucA instead of CRO. Of course, this approach is limited because the fluorescence intensity cannot represent intact ApDCs when degraded *in vivo*. Furthermore, 5′-NucA-SMCC-DM1 and 5′-NucA-SPDMV-DM1 showed potent antitumor efficacy with significantly decreased toxicity to the liver and heart compared with DM1 alone and the corresponding 5′-CRO-SMCC-DM1 and 5′-CRO-SPDMV-DM1. Notably, 5′-NucA-SPDMV-DM1 showed the best tumor size reduction among all the treatment groups, which might be attributed to the potential bystander killing effect with the cleavage of the disulfide bond compared with the noncleavable linker SMCC.

In addition, we further used STRING to verify potential interacting proteins/pathways related to DM1/NucA, and KEGG analysis was further used to identify potential genes that are possibly enriched in these pathways (Figure S11). It is noteworthy that this is generally hypothesis-generating, and further proteomics/genomics data could provide more substantive evidence for the enrichment.

## Conclusion

In this study, we utilized nucleolin aptamer NucA to conjugate DM1 with two types of linkers, aiming to selectively deliver DM1 to pancreatic cancer cells. The results demonstrated that the 5′-linked ApDCs could retain potent cytotoxicity and an identical antitumor mechanism against PANC-1 and MIA PaCa-2 compared with DM1, and they exhibited significantly improved targeting ability in comparison with normal MIHA cells. 5′-NucA-SMCC-DM1 and 5′-NucA-SPDMV-DM1 also exhibited excellent serum stability. Macropinocytosis is likely to be involved in ApDC cellular internalization in PANC-1 cells. 5′-NucA-SMCC-DM1 and 5′-NucA-SPDMV-DM1 demonstrated potent *in vivo* antitumor efficacy with significantly decreased toxicity.

Our findings encompassed the development of NucA-DM1 conjugates with SMCC and SPDMV linkers in the preclinical studies of pancreatic cancer. A deeper investigation of 3′- and 5′-linked differences might offer further insight into the binding mechanism between nucleolin and NucA, as it is not well understood. Certain modifications could also be adopted to increase the binding affinity and, most importantly, stability in the circulatory system. Considering the complicated tumor microenvironment of pancreatic cancer, animal models that can better mimic a real TME could further enhance the targeted delivery by these ApDCs.

## Materials and Methods

### Chemistry

Mertansine (DM1) was obtained from Sigma-Aldrich, cleavable and noncleavable linkers were purchased from MedChemExpress, and all other reagents were purchased from Energy Chemicals and used without further purification. All of the oligonucleotides were purchased from Sangon Biotech. HPLC spectra and HR-MS data of all the synthesized ApDCs can be found in the Supporting Information. Control aptamer CRO (Sequence: TTTCCTCCTCCTCCTTCTCCTCCTCCTCC) was bought from Sangon Biotech.^30,31^

### Synthesis of NucA/Cro-Linker

In brief, an oligonucleotide with a 5′ end amino modifier (0.84 mg, 100 nmol) was dissolved in 100 μL of PB buffer (pH 8), and the linker (5 μmol) cross-linker, freshly dissolved in DMSO (100 μL), was subsequently added. The reaction mixture was stirred at room temperature for 2 h in buffer pH 8. Upon completion, particulate matter was removed by centrifugation at 16,000*g* for 10 min, and the crude NucA-SMCC oligonucleotide was further purified by two methods: ethanol precipitation and reversed-phase HPLC (Agilent 1260 HPLC system along with a UV detector set at 254 nm, XBride Oligonucleotide BEH C18 OBD Prep Column 2.5 μm, 10 mm × 50 mm) using a TEAA/ACN (0.05 M, pH 7.0) system. The ethanol precipitation product was used for the next step directly, with a yield of 80–95%.

### Synthesis of NucA/Cro-Linker-DM1 Conjugates

The synthesis of NucA-DM1 conjugates mainly referred to Tan et al.^32^ Briefly, 100 nmol of SMCC-NH_2_ modified oligonucleotide was dissolved in 1 mL of PB buffer (pH = 7), and a 10-fold molar mass of DM1 was dissolved in 100 μL of DMSO. The solutions were mixed and incubated in a 37 °C shaker (rotation rate: 150 rpm) overnight. The reaction buffer was dried in a freeze centrifuge to eliminate the DMSO. Then, the freeze-dried powder was dissolved using 0.1 M triethylamine-glacial acetic acid buffer (TEAA) and purified by reverse high-performance liquid chromatography (Agilent 1260 HPLC system with a UV detector set at 254 nm, XBride Oligonucleotide BEH C18 OBD Prep Column 2.5 μm, 10 mm × 50 mm) with a mobile phase containing 0.1 M TEAA and acetonitrile. Finally, the products were collected, desalinated, and quantified using a NanoDrop microvolume spectrophotometer set to oligonucleic acid mode with an A260 detection wavelength. Cy5-labeled conjugates were also prepared similarly, using Cy5-SMCC-NH_2_-modified oligonucleotide as the starting material .

The resulting ApDCs were purified by an Agilent 1260 HPLC system equipped with a UV detector set at 254 nm. The chromatographic separation of the ApDCs was achieved on an XBridge Oligonucleotide BEH C18 OBD Prep Column (2.5 μm, 10 mm × 50 mm). Mobile phase components A and B were water with 50 mM TEAA and acetonitrile, respectively. The ApDCs were separated using a gradient elution (0 min, 5% phase B; 30 min, 65% phase B; 40 min, 95% phase B), with a flow rate of 1.2 mL/min and an ambient column temperature. Desalting of the ApDCs was performed using HiTrap Desalting 5 mL columns (×3). The ApDCs were separated using water, with a flow rate of 1.2 mL/min and an ambient column temperature. The NanoDrop microvolume spectrophotometer was set to Oligonucleic Acid mode with an A260 detection wavelength for concentration identification. Each 1 μL sample was loaded onto the NanoDrop UV–vis spectrophotometer. The yields of each conjugate were 50–70%.

LC-MS confirmation of the synthesized ApDCs utilized a Waters ACQUITY UPLC system along with a UV detector set at 254 nm. The chromatographic separation of the ApDCs was achieved on a Waters BEH C18 Column (1.7 μm, 2.1 mm × 100 mm). Mobile phase component A was water with 7 mM TEA and 100 mM HFIP, while mobile phase component B was methanol. The ApDCs were separated using gradient elution (0.5 min, 5% phase B; 5 min, 100% phase B), accompanied by a flow rate of 0.2 mL/min.

### Cell Culture

Human pancreatic epithelioid carcinoma cell line PANC-1 (RRID: CVCL_0480), human pancreatic cancer cell line MIA PaCa-2 (RRID: CVCL_0428), and immortalized human hepatocytes MIHA (RRID: CVCL_SA11) were obtained from the American Type Culture Collection (ATCC, USA) and cultured in Dulbecco’s Modified Eagle Medium (DMEM; Gibco), supplemented with 10% fetal bovine serum (FBS, Gibco) and 100 μg/mL penicillin and streptomycin (Gibco) at 37 °C in a humidified atmosphere comprising 5% CO_2_. All of the cell lines were tested to be mycoplasma-free before experiments (PlasmoTest, Mycoplasma Detection Kit, InvivoGen). Enzyme-Linked Immunosorbent Assay (ELISA) kits were bought from Abcam.

### *In Vitro* Cell Viability Assay

The cytotoxicity of all the synthesized conjugates 3′-NucA-SMCC-DM1, 5′-NucA-SMCC-DM1, 3′-NucA-SPP-DM1, 5′-NucA-SPP-DM1, 3′-NucA-SPD-DM1, 5′-NucA-SPD-DM1, 3′-NucA-SPDMV-DM1, 5′-NucA-SPDMV-DM1, 3′-CRO-SMCC-DM1, 5′-CRO-SMCC-DM1, 3′-CRO-SPP-DM1, 5′-CRO-SPP-DM1, 3′-CRO-SPDP-DM1, 5′-CRO-SPDP-DM1, 3′-CRO-SPDMV-DM1, 5′-CRO-SPDMV-DM1, DM1, and NucA were evaluated using the Cell Counting Kit-8 (CCK8) assay. PANC-1, MIA PaCa-2, and MIHA cells were seeded in 96-well plates with 5 × 10^3^ cells in each well and incubated overnight for adherence. Generally, different drug concentrations ranging from 1 to 500 nM, using a serial 2-fold dilution method, were added into preincubated 96-wells of PANC-1, MIA PaCa-2, and MIHA cells. After 72 h of incubation at 37 °C, CCK8 solution was added to each well. The absorbance was detected at 450 nm after 2 h. EC_50_ values at 72 h were calculated using GraphPad Prism 9 based on the viability curve data.

### Cell Cycle Assay

Cell cycle analysis was performed by following the instructions of the Propidium Iodide Flow Cytometry Kit (Abcam).^18^ Briefly, PANC-1 and MIA PaCa-2 cells (5 × 10^5^ per well) were seeded in 6-well plates and incubated overnight. After washing with PBS, the cells were incubated with 250 nM DM1, 5′-NucA-SMCC-DM1, at 37 °C for 48 h. At the end of the incubation, the cells were trypsinized, washed, and fixed in 66% ethanol on ice. After storage at 4 °C overnight, the cells were washed, resuspended in 200 μL of 1 × (Propidium Iodide + RNase) Staining Solution, and incubated at 37 °C in the dark for 30 min. Finally, DNA content was measured by Flow Cytometry (BD 831 Immunocytometry Systems); the percentage of cells in each phase of the cell cycle was calculated using the ModFit software.

### Serum Degradation Assay

Serum degradation assays were performed by incubating 5′-NucA-SMCC-DM1, 5′-NucA-SPP-DM1, 5′-NucA-SPDP-DM1, and 5′-NucA-SPDMV-DM1 (0.4 nmol, 2 μL) in normal human serum (8 μL) at 37 °C in a metal bath with heated lids. After treatment for various periods of time, the solution was immediately frozen in a −80 °C freezer. Afterward, the addition of 2 × loading buffer (10 μL) was carried out, denatured, and analyzed on 20% polyacrylamide gels (110 V, 2 h). Finally, the gels were visualized by 0.03% Gel Green staining and analyzed using Image Lab software.

### Cellular-Bound and Internalized Conjugates

PANC-1 and MIA PaCa-2 cells were seeded in a 24-well plate at a density of 2 × 10^5^ cells per well and incubated overnight. For the concentration-dependent uptake assay, the final concentrations of 3′-Cy5–5′-NucA-SMCC-DM1, 3′-Cy5–5′-NucA-SPDMV-DM1, 3′-Cy5-5′-CRO-SMCC-DM1, and 3′-Cy5-5′-CRO-SPDMV-DM1 were 0 nM, 31.25 nM, 62.5 nM, 125 nM, and 250 nM, respectively. After 2 h of incubation, the cells were washed three times with PBS, trypsinized, and resuspended in 400 μL PBS after centrifugation (1000 rpm, 5 min). PANC-1 and MIA PaCa-2 cells without any drug incubation were used as a blank control to measure background signals, which were subtracted from the final calculations. The experiments were performed in triplicates and repeated three times. The fluorescence was measured by Flow Cytometry (BD 831 Immunocytometry Systems). The data obtained were further processed using the FlowJo software to depict the curve and calculate the mean or median fluorescence intensity (MFI), with the zero-drug concentration as the blank control. To distinguish the surface-bound and internalized conjugates, an additional step involving DNase treatment (0.1 μg/mL, 30 min) was included for comparison.

### Confocal Imaging for Endocytosis Pathways

Confocal imaging for endocytosis pathway investigation referred to Li et al.^18^ PANC-1 or MIA PaCa-2 cells were seeded in glass-bottomed confocal dishes at a density of 5 × 10^4^ per well and incubated overnight. Cells were then incubated with 250 nM of 3′-Cy5-5′-NucA-SMCC-DM1 or 3′-Cy5-5′-CRO-SMCC-DM1 and Alexa Fluor 488-labeled endocytic markers (50 μg/mL dextran, 50 μg/mL transferrin, and 5 μg/mL CTX-B) at 37 °C for 2 h. A volume of 4 μg/mL Hoechst 33,342 was then added during the final 15 min of the incubation. After 2 h of incubation, the cells were washed and visualized using a Leica SP5 X laser scanning confocal microscope.

For chemical inhibition of endocytosis pathways, PANC-1 cells were plated in 6-well plates at a density of 5 × 10^5^ cells per well and allowed to adhere overnight. Cells were preincubated for 30 min with the macropinocytosis inhibitor EIPA at specified concentrations. Following pretreatment, the NucA-DM1 conjugate was introduced at 500 nM. After 2 h, cells were trypsinized and centrifuged (1000 rpm, 5 min), and the supernatant was discarded. The cell pellet was washed twice with PBS and finally resuspended in 400 μL of PBS. Fluorescence was quantified by flow cytometry on a BD FACScan instrument, collecting 10,000 events per sample. Background fluorescence, determined from DMSO-treated control cells, was subtracted from the experimental values.

### Animal Handling

All animal experiments were approved by the Ethics Committee of Hong Kong Baptist University (approval number: REC/24-25/0038). Eight-week-old female BALB/c nude mice were purchased from the Laboratory Animal House of Hong Kong Baptist University. All mice were housed in the Laboratory Animal House of Hong Kong Baptist University. The animal house is temperature-controlled with a 12-h light/dark cycle. Food and water were available ad libitum. At least a week’s adaptation was given to the mice before starting any experiments. The procedures for all *in vivo* studies have gained ethics approval from the Animal Experimentation Ethics Committee of Hong Kong Baptist University.

### *In Vivo* Antitumor Efficacy

Eight-week-old female BALB/c nude mice were inoculated subcutaneously with 2 × 10^6^ PANC-1 cells in the left armpit. Tumors were observed 2 weeks after inoculation. The tumor-bearing nude mice were randomly divided into groups, with six mice in each group, for further studies. NucA, DM1, 5′-NucA-SMCC-DM1, 5′-NucA-SPDMV-DM1, 5′-CRO-SMCC-DM1, and 5′-CRO-SPDMV-DM1, at a dosage with the equivalent DM1 concentration of 0.1 mg/kg, were given to mice of 4 groups by i.v. twice a week for 4 weeks. Another group was given vehicle solution PBS as the control group. The tumor size and body weight were monitored every 4 days. The tumor size was measured using a caliper, and the tumor volume was calculated using the formula *V* = 1/2 × L × W^2^.

### Biochemical Assays for Liver and Cardiac Function Enzymes

At the end of the treatment (day 28), the mice were killed, and blood was collected for biochemical analysis. The levels of two liver function enzymes, aspartate aminotransferase (AST) and alanine aminotransferase (ALT), were analyzed using ELISA kits (Abcam). The levels of two cardiac function enzymes, creatine phosphokinase (CPK) and creatine kinase myocardially bound (CK-MB), were analyzed using the Creatine Kinase Activity Assay Kit (Abcam) and the Mouse Creatine Kinase MB isoenzyme (CK-MB) ELISA Kit (Cusabio Biotech), respectively.

### Statistics

Statistical analysis of the experimental results was performed using GraphPad Prism 9 (GraphPad Software, Inc., La Jolla, CA, USA). All statistics are presented as the mean ± SD from at least three independent experiments. Unpaired Student’s *t*-test was used to assess differences between two groups, while analysis of variance (ANOVA) was used for comparisons among multiple groups. *p* < 0.05 was considered to indicate significant differences.

## Supplementary Material



## Data Availability

The data underlying this study is available in the published article and its Supporting Information.

## Notes

Surface plasmon resonance, plasma half-life, DM1 release, *in vivo* biodistribution, STRING database analysis and pathway enrichment, HPLC spectra, and HR-MS of conjugates (PDF)

## Abbreviations

ADC: antibody–drug conjugate
ALT: alanine aminotransferase
AST: aspartate aminotransferase
ApDC: aptamer–drug conjugate
CD33: sialic acid-binding Ig-like lectin 3
CK-MB: creatine kinase myocardial bound
CPK: creatine phosphokinase
Cy5: sulfo-cyanine5
DM1: mertansine
EIPA: 5-(N-ethyl-N-isopropyl)­amiloride
ELISA: enzyme-linked immunosorbent assay
HER2: human epidermal growth factor receptor 2
HRMS: high-resolution mass spectrum
KEGG: Kyoto encyclopedia of genes and genomes
NucA: antinucleolin aptamer AS1411
PANC-1: human pancreatic epithelioid carcinoma
MIA PaCa-2: human pancreas epithelial carcinoma
MIHA: normal human liver cells
MTS: maytansine
PC: pancreatic cancer
SMCC: N-succinimidyl 4-(N-maleimidomethyl)­cyclohexane-1-carboxylate
SPP: N-succinimidyl 4-(2-pyridyldithio)­pentanoate
SPDMV: N-succinimidyl 4-methyl-4-(2-pyridyldithio)­pentanoate
SPDP: N-succinimidyl 3-(2-pyridyldithio)­propanoate
S.D.: standard deviation
