# Supplementary material for: 5′-NucA-SMCC-DM1 and 5′-NucA-SPDMV-DM1 are Potent Aptamer–Drug Conjugates against Pancreatic Cancer
Source: ACS Omega. 2026 Feb 3;11(7):12461–71. doi: 10.1021/acsomega.5c11364 (PMC12947138; doi:10.1021/acsomega.5c11364)
Supplement: Supplementary file 1 [file ao5c11364_si_001.pdf]

SUPPORTING INFORMATION

5′-NucA-SMCC-DM1 and 5′-NucA-SPDMV-DM1 are potent aptamer-drug conjugates against pancreatic cancer

Hong Dai[1#], Razack Abdullah[2#], Wenqiong Huang[3], Xiaoli Chen[3], Aiping Lu[3], Kenneth CP Cheung[3*]

[1] Department of Chemistry, The Hong Kong University of Science and Technology, Clear Water Bay, Kowloon, Hong Kong SAR, China
[2] Law Sau Fai Institute for Advancing Translational Medicine in Bone and Joint Diseases, School of Chinese Medicine, Hong Kong Baptist University, Kowloon, Hong Kong SAR, China
[3] Phenome Research Center, School of Chinese Medicine, Hong Kong Baptist University, Hong Kong, China

[#] These authors contributed equally.
* Correspondence: kcpcheung@hkbu.edu.hk

Keywords: Aptamer-drug conjugate, NucA, Pancreatic cancer, Mertansine, DM1

Table of Contents

Materials and methods
Figure S1-S9, HPLC spectra and HR-MS of synthesized conjugates (**Figure S10-S31**)

Materials and Methods

Cell culture

Human pancreatic epithelioid carcinoma cell line PANC-1 (RRID: CVCL_0480), human pancreatic cancer cell line MIA PaCa-2 (RRID: CVCL_0428), immortalized human hepatocytes MIHA (RRID: CVCL_SA11) were obtained from American Type Culture Collection (ATCC, USA) and cultured in Dulbecco's Modified Eagle Medium (DMEM; Gibco), supplemented with 10% fetal bovine serum (FBS, Gibco), and 100 μg/mL penicillin & streptomycin (Gibco) at 37℃ in a humidified atmosphere comprising 5% CO_2.

In vitro cell viability assay

The cytotoxicity of 5′-NucA-SMCC-DM1, 5′-NucA-SPDMV-DM1, DM1 and NucA against cocultured PANC-1 and MIHA were evaluated using Cell Counting Kit-8 (CCK8) assay. PANC-1 and MIHA cells (1:1) were co-seeded in 96-well plates with 5×10^3 cells in each well and incubated overnight for adherence. Generally, different drug concentrations ranging from 1-500 nM using a serial twofold dilution method were added into pre-incubated 96-well. After incubation of 72 h at 37°C, CCK8 solution was added to each well. The absorbance was detected at 450 nm after 2 h. EC_{50} values of 72 h were calculated using GraphPad Prism 9 based on the viability curve data. For the CRO-based conjugates, the protocols are adopted without further modifications.

DM1 release in PANC-1 cells

PANC-1 cells were pre-seeded into 6-well plates at a density of 5×10^5 cells/well in 2 mL of complete media and cultured for 24 h. 5′-NucA-SMCC-DM1 and 5′-NucA-SPDMV-DM1 (0.2 nmol) were added and cocultured. Cells were harvested at timed intervals (0-24 h) and the media was also collected. Cells were washed by ice-cold PBS and trypsinized. 1 ml cold acetonitrile was added, mixed with media and the mixture was vortexed and cultured on ice for 30 min. The resulting mixture was centrifuged (14,000 rpm, 10 min) and clear supernatant was collected, lyophilized for further LC-MS analysis. The remaining percentage of intact conjugates was calculated based on the calibration curve. LC-MS utilized a Waters ACQUITY UPLC system along with UV detector set at 254 nm with Waters ®BEH C18 Column (1.7 μm, 2.1 mm×100 mm). Data were measured in triplicate and plotted by GraphPad Prism 9. S.D., standard deviation.

Surface plasmon resonance (SPR)

The interaction between NucA-DM1 conjugates and nucleolin was characterized by SPR on a GE Biacore X100 instrument. The nucleolin protein was covalently immobilized on a CM5 sensor chip via standard amine-coupling chemistry. Initial surface activation was achieved using a 1:1 mixture of 0.5 M NHS and 0.1 M EDC, after which nucleolin in 10 mM sodium acetate (pH 5.0) was introduced for conjugation. Analytes (NucA, 5′-NucA-SMCC-DM1 and 5′-NucA-SPDMV-DM1) were prepared in a concentration series from to 31.25 nM to 1 μM in 1× PBS buffer. Each analyte concentration was injected in duplicate over the functionalized surface to verify binding reproducibility. A regeneration step with 50 mM

NaOH was applied after each analysis cycle to ensure a uniform surface for subsequent injections. The equilibrium dissociation constant (K_d) was derived from global fitting of the concentration-dependent sensorgrams.

Plasma half-life

Mice were administered a single intravenous injection (equivalent to 0.2 mg/kg DM1) of 3′-Cy5-5′-NucA-SMCC-DM1 or 3′-Cy5-5′-NucA-SPDMV-DM1 via the tail vein. Plasma samples were obtained at predetermined time points, and fluorescence was quantified. The 0-hour time point was modeled by spiking the equivalent dose into naive mouse plasma. The plasma concentration-time curve was constructed based on relative fluorescence units, and the elimination half-life ($t_{1/2}$) was calculated by linear regression of the log-transformed data from the terminal phase.

Animal study for biodistribution in vivo

A biodistribution study was conducted in a mouse xenograft model. BALB/c nude mice were inoculated with 2×10^6 PANC-1 cells, and tumors were allowed to develop for 2 weeks. Tumor-bearing mice were then randomly divided into four groups (n = 6) and treated via subcutaneous injection with 3′-Cy5-5′-NucA-SMCC-DM1, 3′-Cy5-5′-NucA-SPDMV-DM1, 3′-Cy5-5′-CRO-SMCC-DM1 and 3′-Cy5-5′-CRO-SPDMV-DM1 (equivalent to 0.2 mg/kg DM1). At 4 hours post-administration, the mice were euthanized. The heart, liver, spleen, lung, kidney, and tumor were collected and immediately imaged ex vivo using a Maestro 2 Imaging Station (CRI, MA, USA).

STRING Database Analysis and Pathway Enrichment

Using the analytical results from the STRING database, we successfully identified genes (NCL, SCO2, FLT1, KDR, FLT4, MAPT) that exhibit strong interactions with mertansine-targeted genes, all with interaction scores of at least 0.4. These genes, in conjunction with their target genes, formed a network comprising 1,607 genes. Furthermore, we conducted a KEGG pathway enrichment analysis of these genes. This analysis revealed a significant enrichment in the "Cell Cycle" pathway, with a P-value of less than 0.0003, which includes 36 genes that interact with the DM1-targeted genes. Additionally, the "Endocytosis" pathway also showed a notable enrichment trend, with a P-value of 0.0246, involving 43 interacting genes. These findings are mainly hypothesis-based and require more substantial evidence for verification.

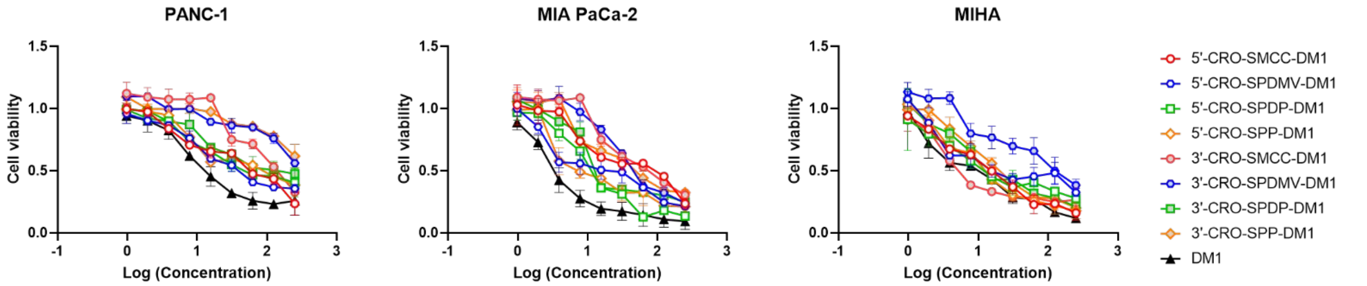

Figure S1. Antiproliferative activity of synthesized control aptamer CRO-drug conjugates in PANC-1, MIA PaCa-2 and MIHA cells. Data were mean ± S.D. of three independent experiments, and each was measured in triplicate (n=3). S.D., standard deviation.

Table S1. EC_{50} values of synthesized control aptamer CRO-drug conjugates against PANC-1, MIA PaCa-2 and MIHA cells.

**Entry**	**EC_{50} (nM, 72 h)**					
	**PANC-1**		**MIA PaCa-2**		**MIHA**	
DM1	8.83 ± 0.73		2.50 ± 0.82		2.70 ± 0.64	
**Linker**	**5′-CRO-DM1**	**3′-CRO-DM1**	**5′-CRO-DM1**	**3′-CRO-DM1**	**5′-CRO-DM1**	**3′-CRO-DM1**
SMCC	31.09 ± 0.97	76.3 ± 0.94	14.59 ± 0.73	25.16 ± 0.60	3.65 ± 0.95	4.63 ± 0.70
SPDP	10.37 ± 0.64	14 ± 0.55	12.53 ± 0.87	9.67 ± 0.70	5.08 ± 0.72	8.56 ± 0.84
SPP	9.87 ± 0.66	255.8 ± 0.84	20.87 ± 0.91	14.09 ± 0.83	6.63 ± 0.93	10.82 ± 0.88
SPDMV	13.41 ± 0.66	79.9 ± 0.66	9.2 ± 0.84	27.62 ± 0.86	2.71 ± 0.81	17.94 ± 0.75

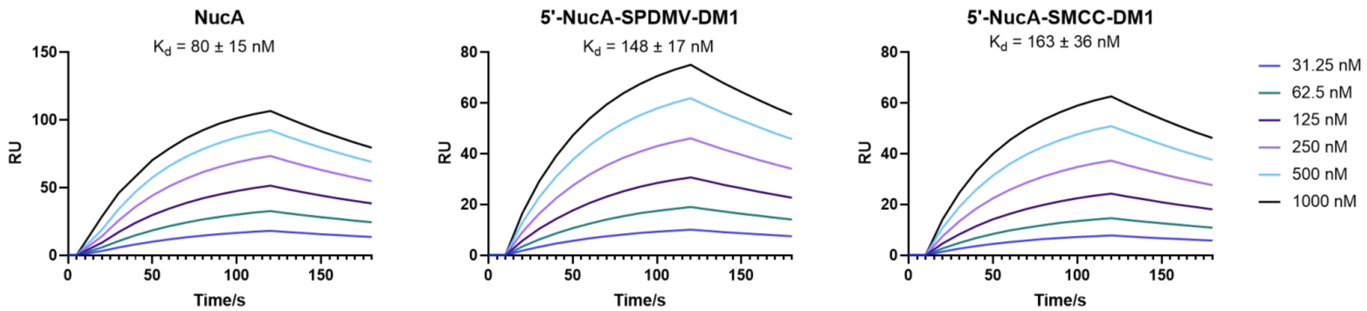

Figure S2. The binding affinity of nucleolin with unmodified NucA, 5′-NucA-SMCC-DM1 and 5′-NucA-SPDMV-DM1, respectively by SPR. RU represented resonance units.

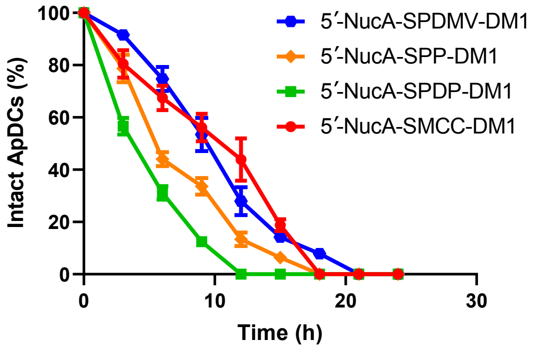

Figure S3. The remaining percentage of synthesized ApDCs after co-incubation within PANC-1 cells. The percentage was calculated by LC-MS at timed intervals (0, 3, 6, 9, 12, 15, 18, 21, 24 h). Data were mean ± S.D. of three independent experiments, and each was measured in triplicate (n=3). S.D., standard deviation.

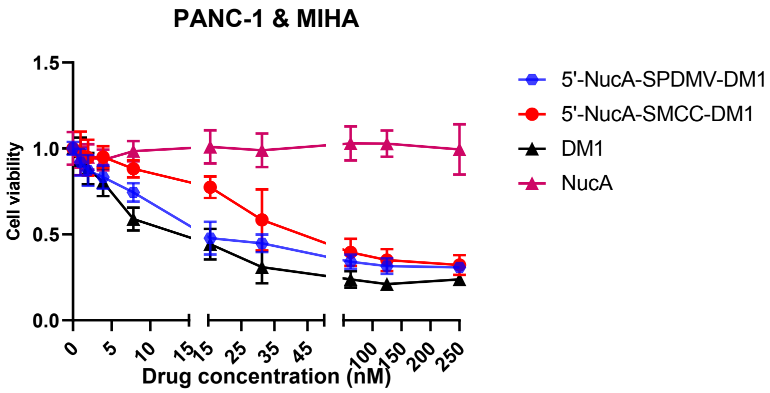

Figure S4. Antiproliferative activity and EC_{50} values of 5′-NucA-SMCC-DM1 and 5′-NucA-SPDMV-DM1 in cocultured PANC-1 and MIHA cells.

Table S2. EC_{50} values of synthesized aptamer conjugates against cocultured PANC-1and MIHA cells.

Entry	EC_{50} (nM)		
	PANC-1 & MIHA	PANC-1	MIHA
DM1	8.07 ± 0.89	7.79 ± 0.84	2.70 ± 0.64
5′-NucA-SMCC-DM1	30.31 ± 0.82	23.14 ± 0.67	44.03 ± 1.23
5′-NucA-SPDMV-DM1	10.52 ± 0.75	10.18 ± 0.61	16.42 ± 0.92
NucA	N/A	N/A	N/A

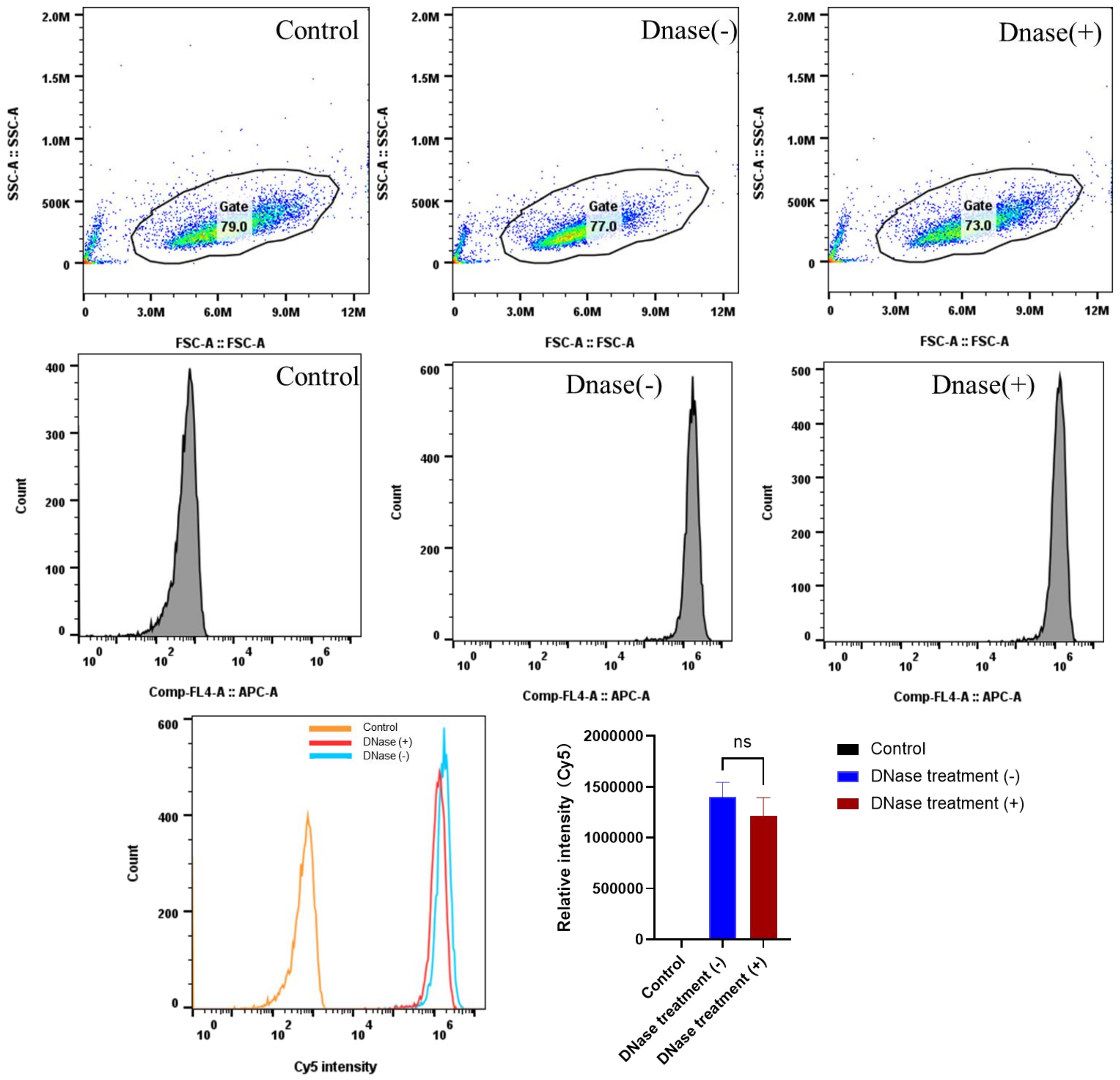

Figure S5. The effect of DNase treatment on cellular internalization. 500 nM 3′-Cy5-5′-NucA-SMCC-DM1 was incubated in PANC-1 cells. After cell harvest, additional DNase treatment was adopted to remove surface-bound conjugates. The resuspended cells were analyzed by flow cytometry. Gating strategy and histograms are exhibited and overlapped. Error bars indicate mean ± S.D. (n = 3 per group). One-way ANOVA was used for statistical analysis, and the significance levels were indicated as *p < 0.05, **p < 0.01, ***p < 0.001, ****p < 0.0001. ns, no significance. S.D., standard deviation.

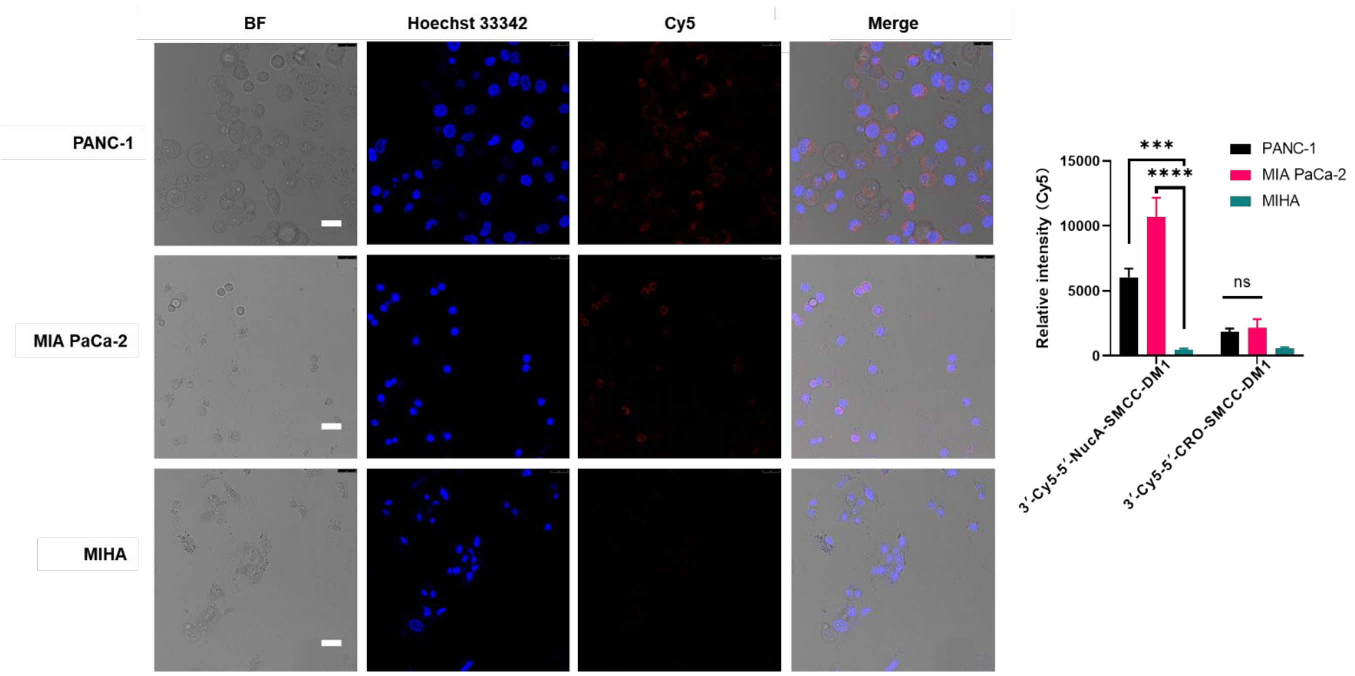

Figure S6. The effect of conjugated DM1 on bound and internalization. 250 nM 3′-Cy5-5′-CRO-SMCC-DM1 (red) was incubated with PANC-1, MIA PaCa-2 and MIHA cells, respectively at 37℃ for 2 h. The nuclei were counterstained with Hoechst 33,342 (blue). Scale bar, 25 μm (the upper right black bar is the original bar and the white bar is for better visualization). Error bars indicate mean ± S.D. (n = 5 per group). Each replicate is from one biological experiment, quantified with 10 independent fields of view. One-way ANOVA was used for statistical analysis, and the significance levels were indicated as *p < 0.05, **p < 0.01, ***p < 0.001, ****p < 0.0001. ns, no significance. S.D., standard deviation.

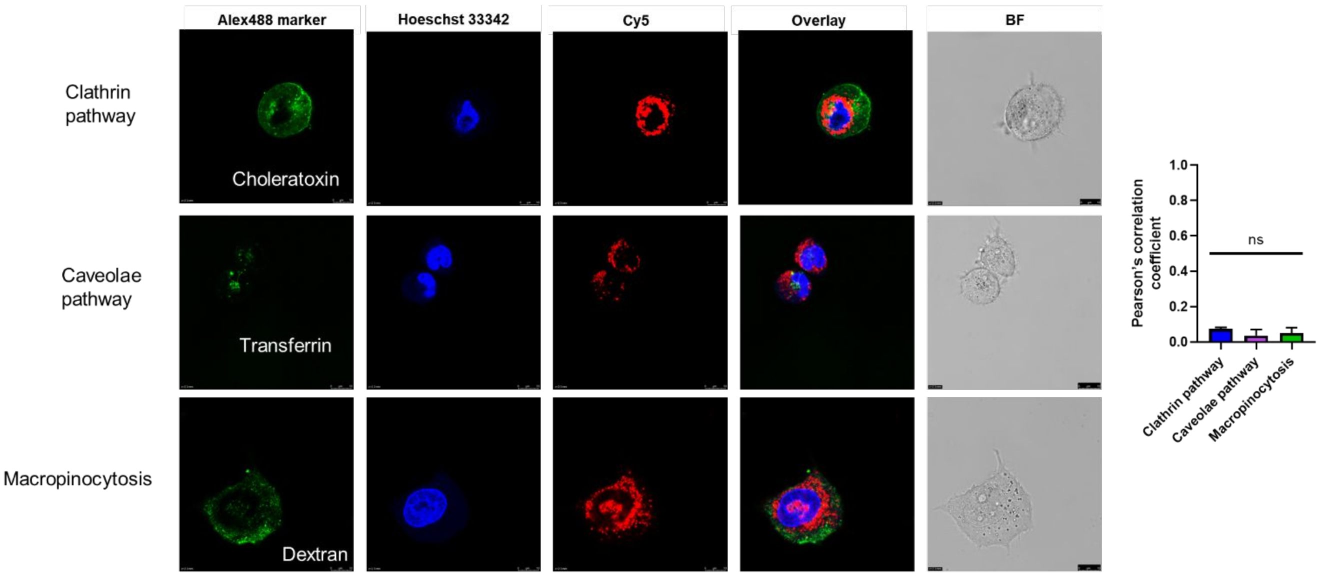

Figure S7. The effect of conjugated DM1 on cellular bound and internalization. 250 nM 3′-Cy5-5′-CRO-SMCC-DM1 was incubated with three Alexa Fluor 488-labled endocytic markers (transferrin, choleratoxin and dextran; green) in PANC-1 cells and the nuclei were counterstained with Hoechst 33,342 (blue). Scale bar, 10 μm (lower right black bar). Error bars indicate mean ± S.D. (n = 5 per group). Each replicate is from one biological experiment, quantified with 10 independent fields of view. One-way ANOVA was used for statistical analysis, and the significance levels were indicated as *p < 0.05, **p < 0.01, ***p < 0.001, ****p < 0.0001. ns, no significance. S.D., standard deviation.

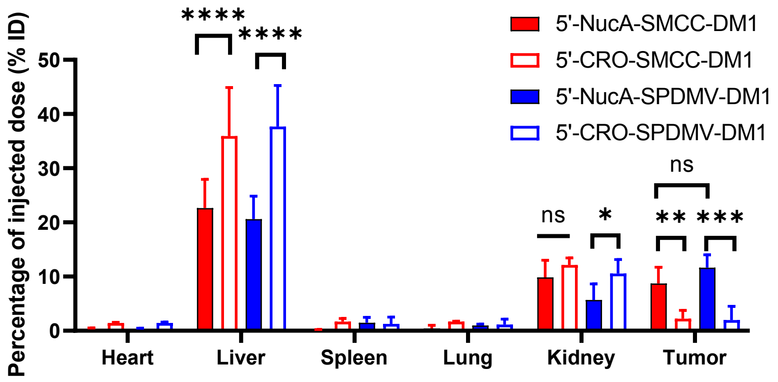

Figure S8. Percentage of injected dose in tumor and major viscera after intravenous injection of 3′-Cy5-5′-NucA-SMCC-DM1, 3′-Cy5-5′-NucA-SPDMV-DM1 and 3′-Cy5-5′-CRO-SMCC-DM1, 3′-Cy5-5′-CRO-SPDMV-DM1 evaluated by measuring the fluorescent intensity of Cy5. The data were presented as the means ± standard deviation. n = 6. One-way ANOVA were used for statistical analysis, and the significance levels were indicated as *$p < 0.05$, **$p < 0.01$, ***$p < 0.001$, ****$p < 0.0001$. ns, no significance. S.D., standard deviation

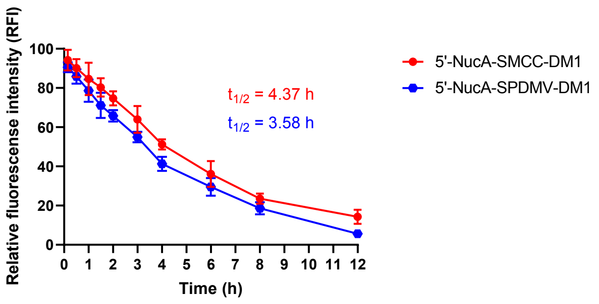

Figure S9. Plasma clearance curve and half-life of 3′-Cy5-5′-NucA-SMCC-DM1 and 3′-Cy5-5′-NucA-SPDMV-DM1. The concentrations of remaining conjugates were measured after a single dose intravenously by detecting the Cy5 fluorescence intensity and standardized to the initial concentration (n = 6). Error bars indicate mean ± standard deviation.

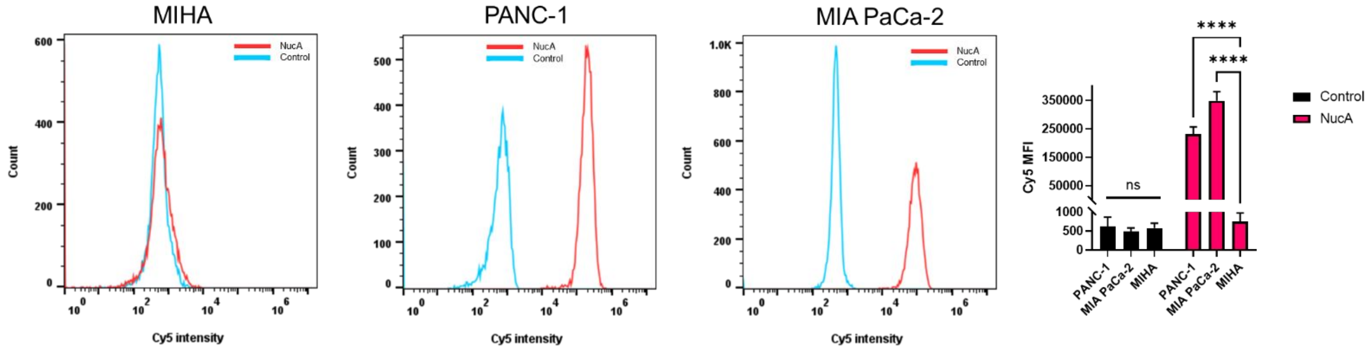

Figure S10. Cy5 fluorescent intensity of 3′-Cy5-NucA measured by flow cytometry after 2 h co-incubation with MIHA, PANC-1 and MIA PaCa-2 cells. Data were mean ± S.D. of three independent experiments (n=3), and each was measured in triplicate. One-way ANOVA were used for statistical analysis, and the significance levels were indicated as *$p < 0.05$, **$p < 0.01$, ***$p < 0.001$, ****$p < 0.0001$. ns, no significance. MFI, mean fluorescence intensity.

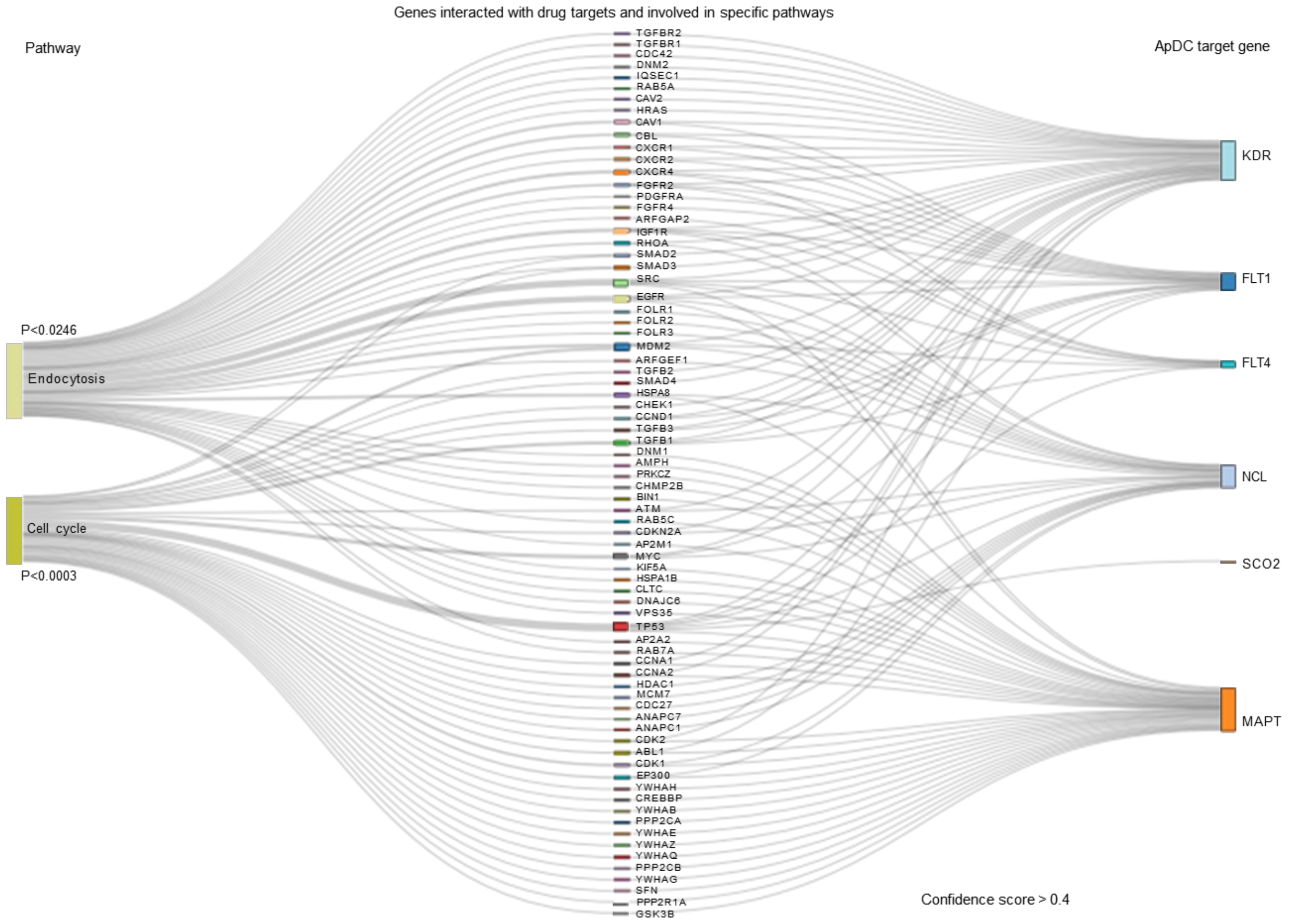

Figure S11. KEGG pathway enrichment analysis. DM1 targeted genes (NCL, SCO2, FLT1, KDR, FLT4, MAPT) and pathway correlations were selected by the components DM1 and NucA. Interacting genes with interaction scores ≥ 0.4 associated with the targeted genes of DM1 were profiled by KEGG analysis.

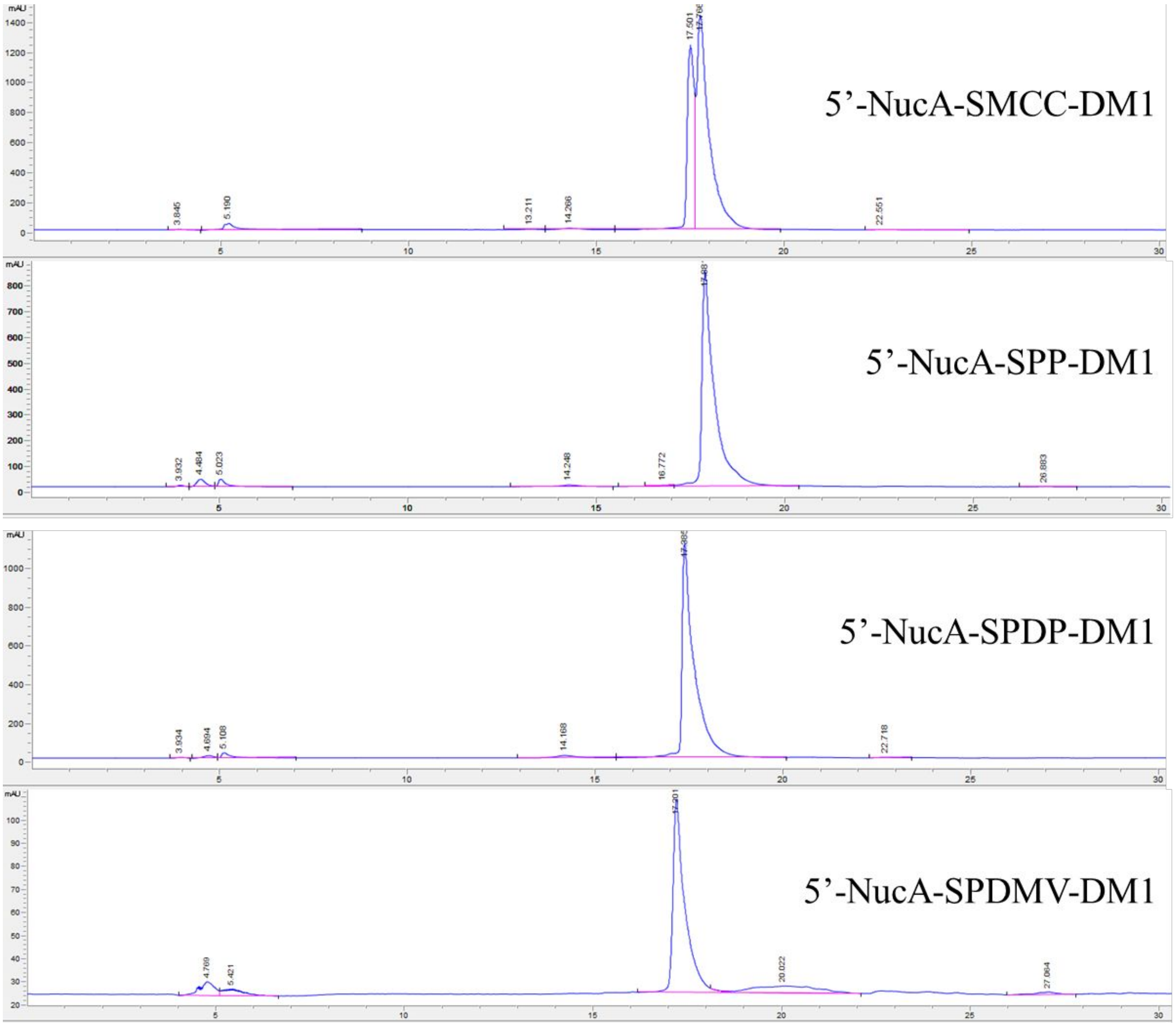

Figure S12. HPLC profiles of representatives of 5′-NucA-DM1 conjugates.

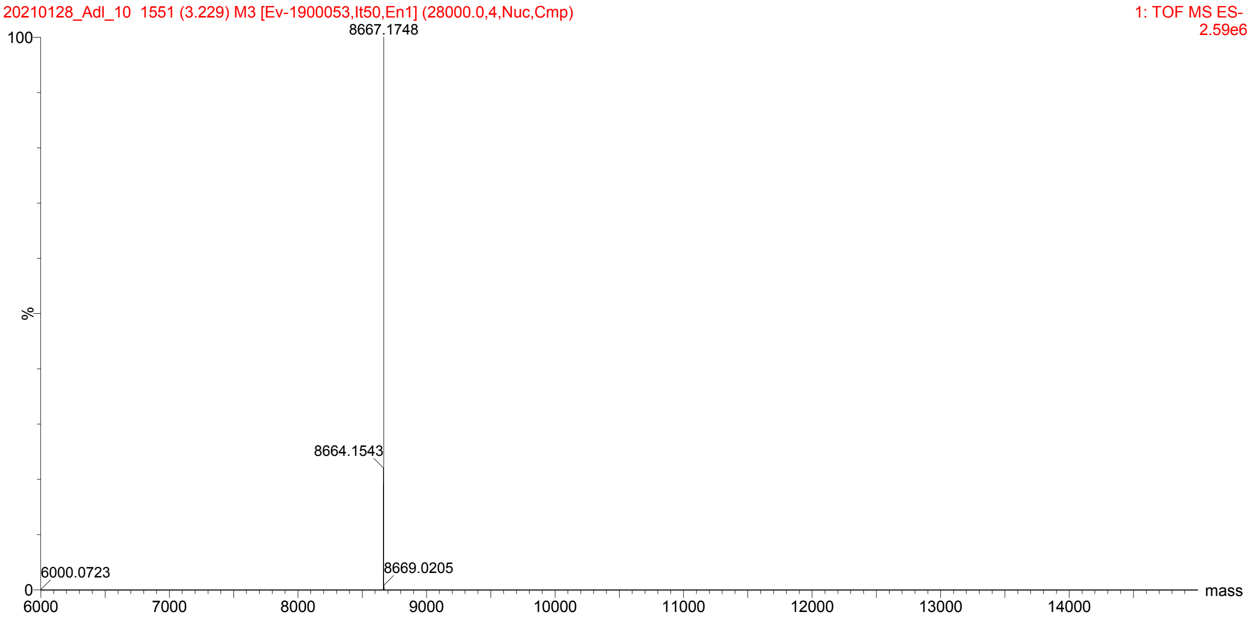

Figure S13. HR-MS of 5′-Amino As1411-SMCC conjugate.

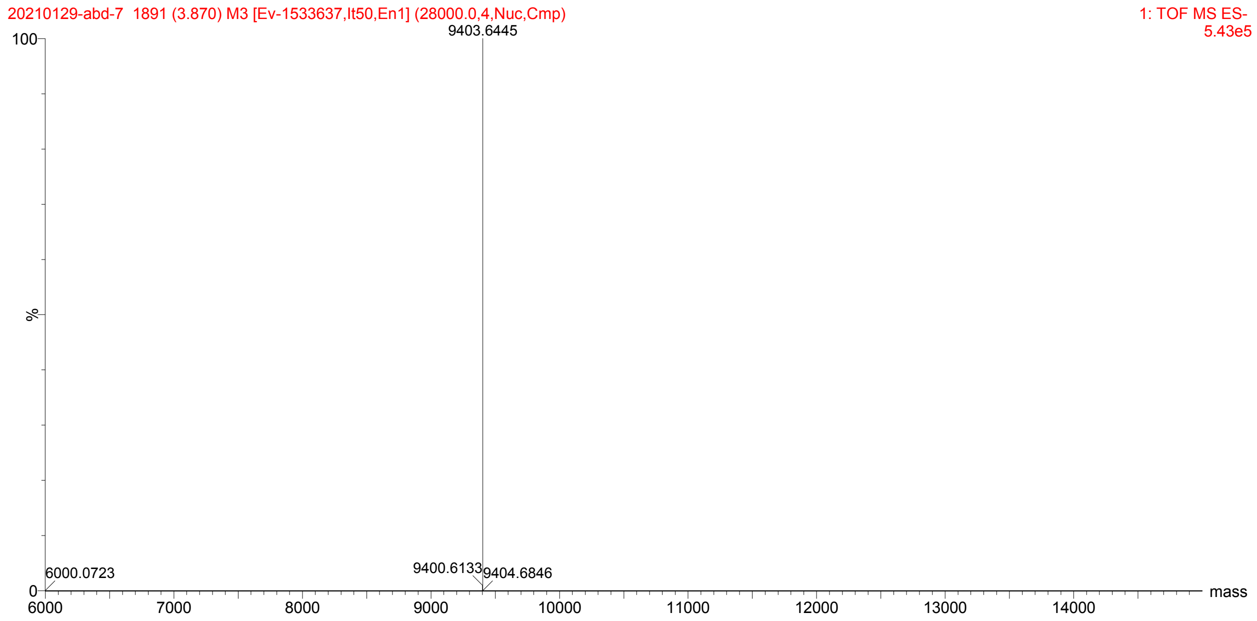

Figure S14. HR-MS of 5′-NucA-SMCC-DM1 conjugate.

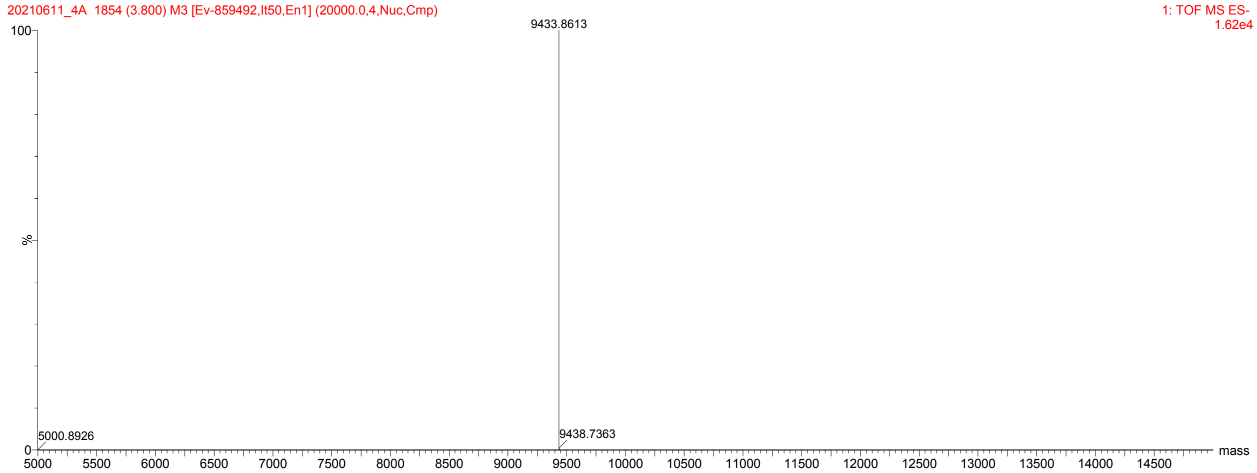

Figure S15. HR-MS of 5′-NucA-SMCC-DM1 conjugate.

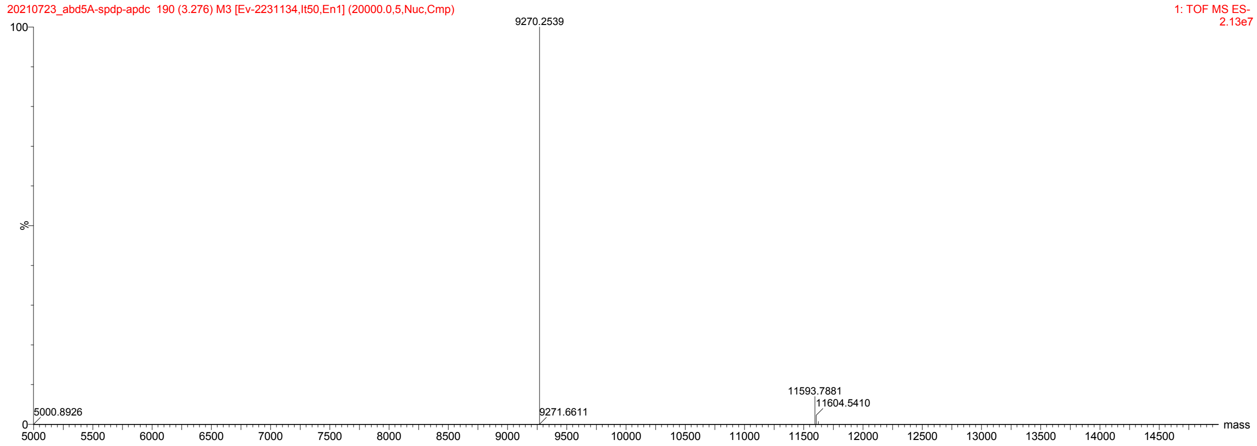

Figure S16. HR-MS of 5′-NucA-SPDP-DM1 conjugate.

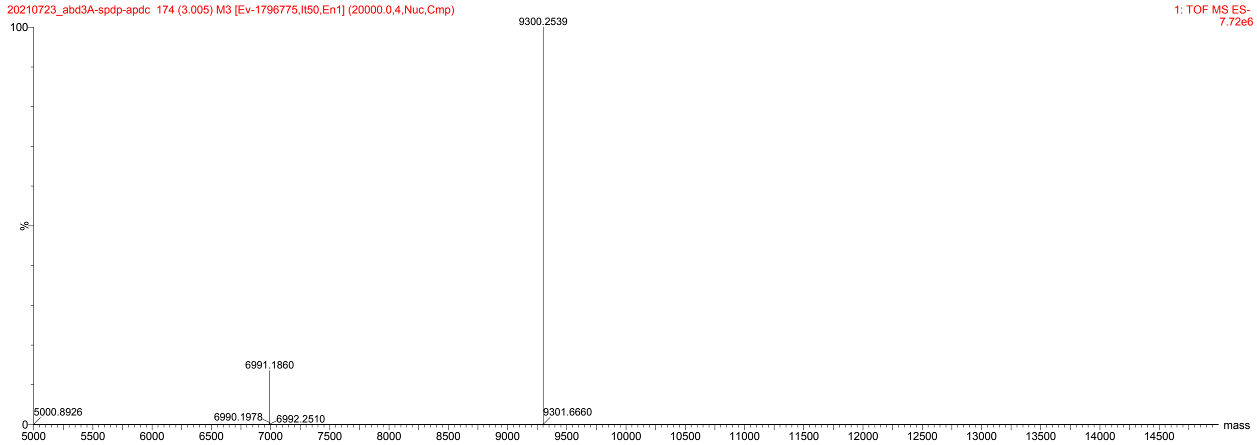

Figure S17. HR-MS of 3′-NucA-SPDP-DM1 conjugate.

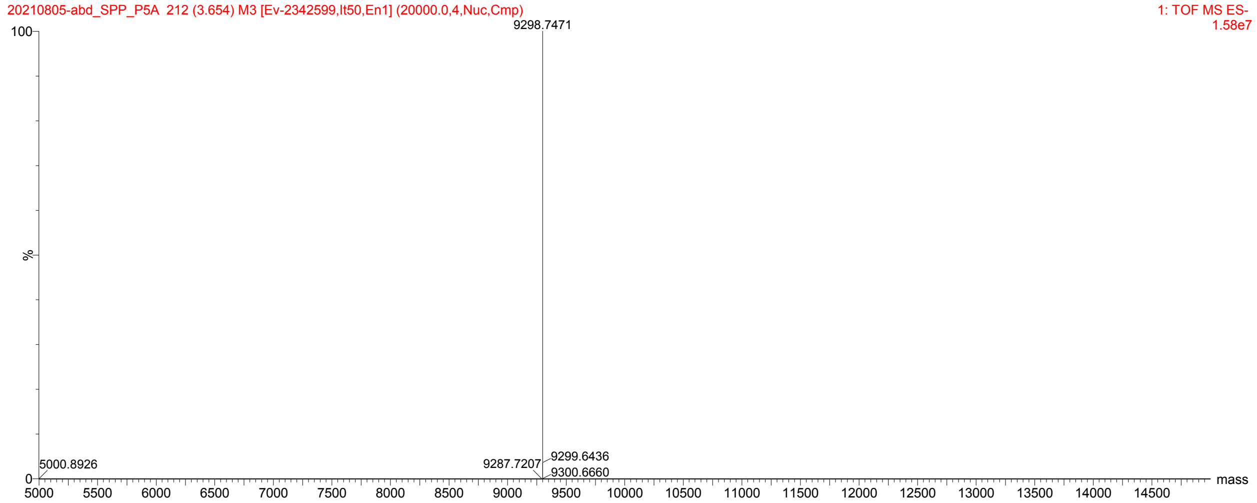

Figure S18. HR-MS of 5′-NucA-SPP-DM1 conjugate.

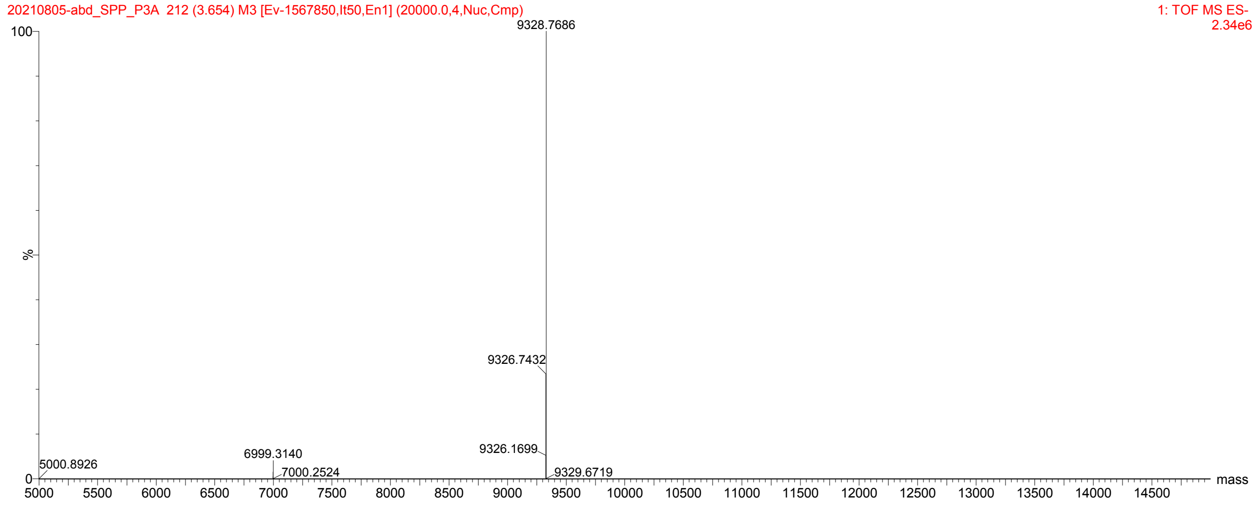

Figure S19. HR-MS of 3′-NucA-SPP-DM1 conjugate.

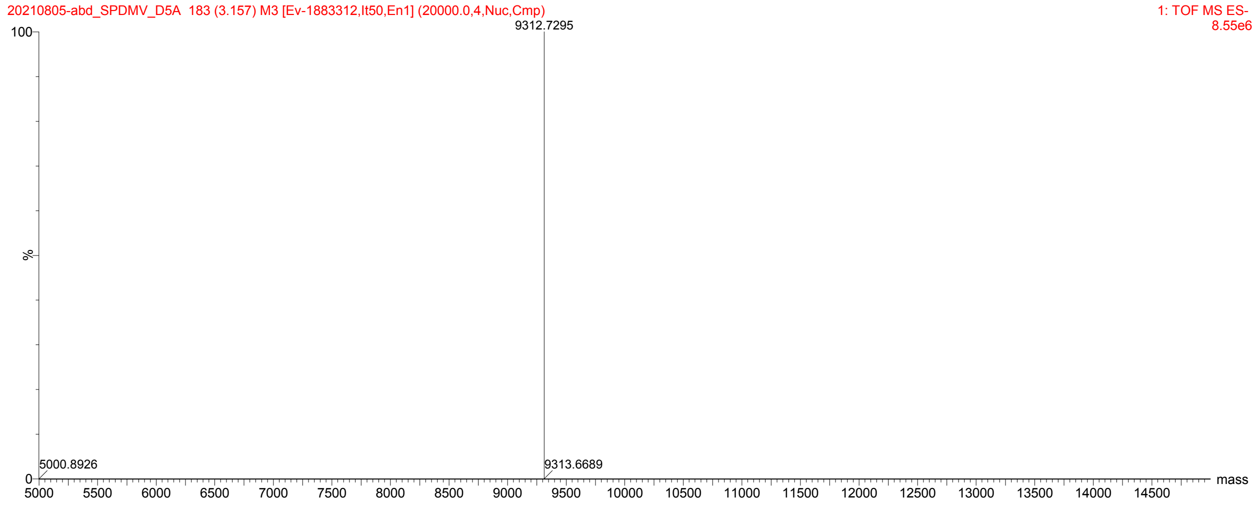

Figure S20. HR-MS of 5′-NucA-SPDMV-DM1 conjugate.

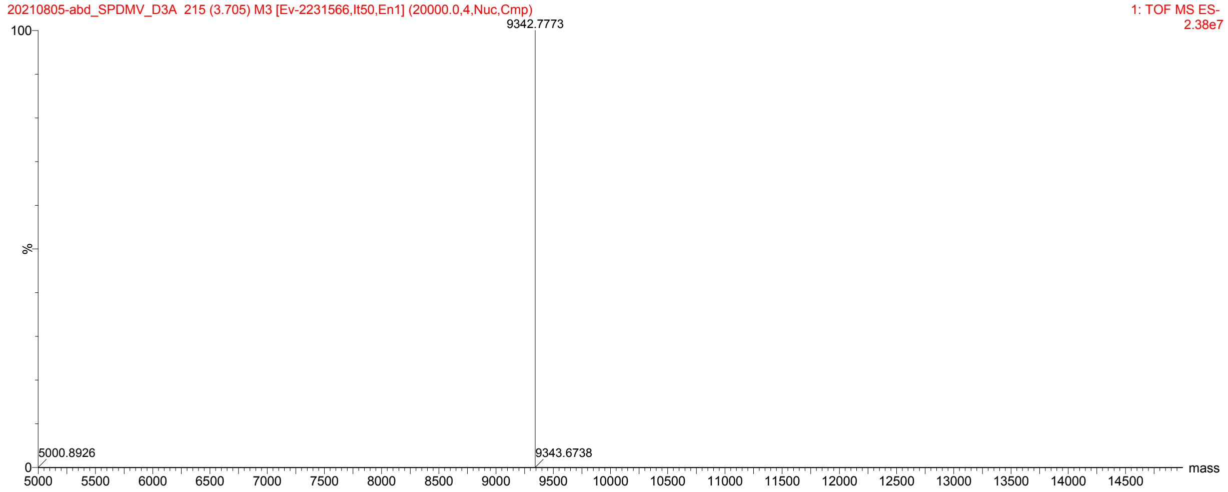

Figure S21. HR-MS of 3′-NucA-SPDMV-DM1 conjugate.

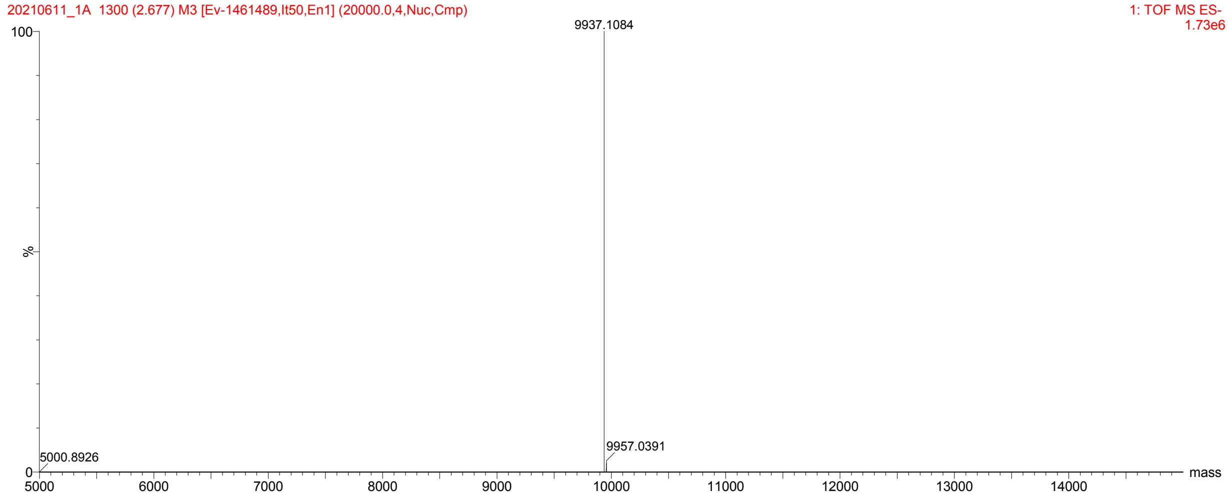

Figure S22. HR-MS of 3′-Cy5-5′-NucA-SMCC-DM1 conjugate.

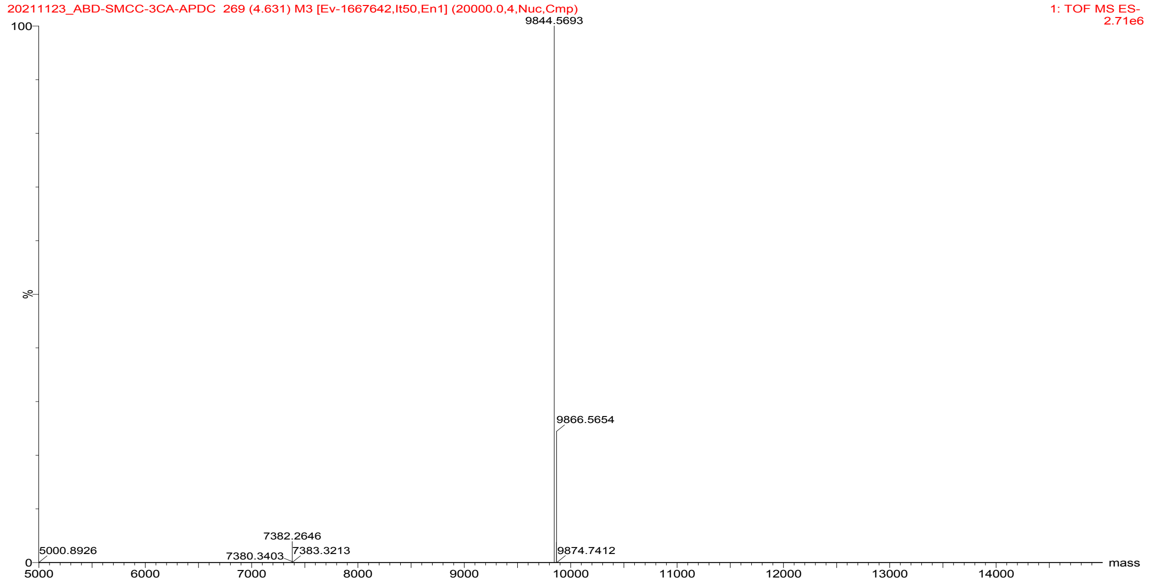

Figure S23. HR-MS of 3′-Cy5-5′-NucA-SPDMV-DM1 conjugate.

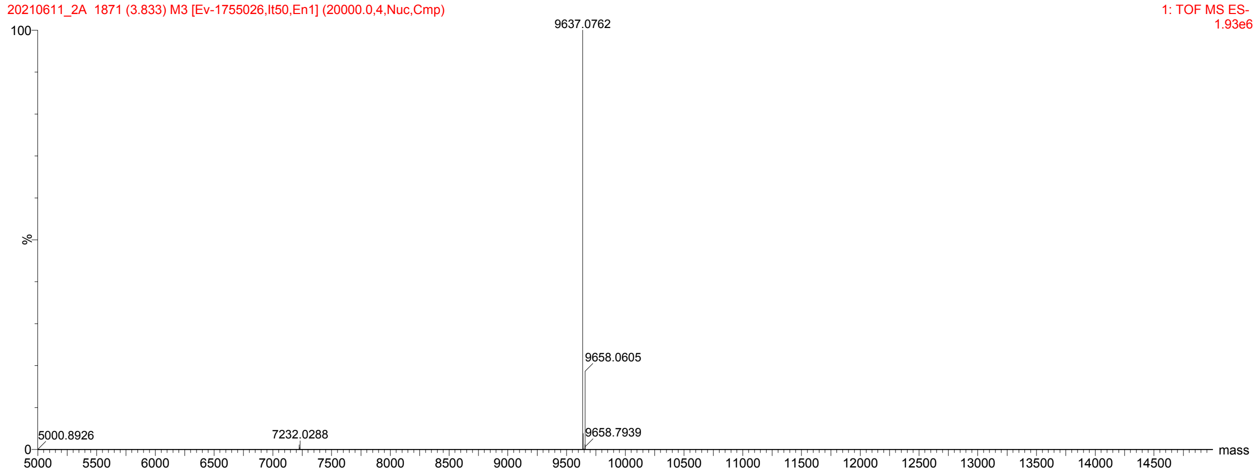

Figure S24. HR-MS of 5′-CRO-SMCC-DM1 conjugate.

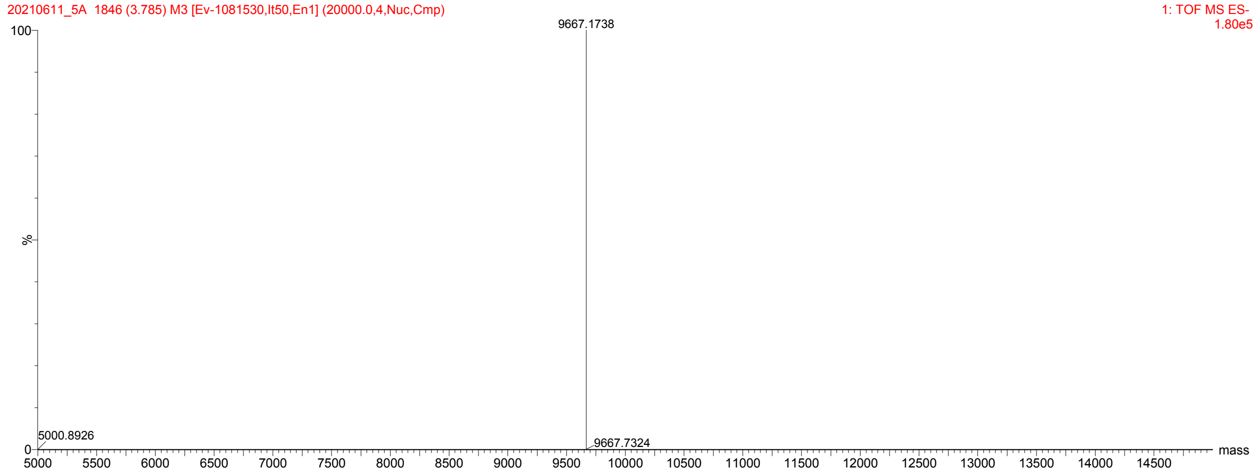

Figure S25. HR-MS of 3′-CRO-SMCC-DM1 conjugate.

189

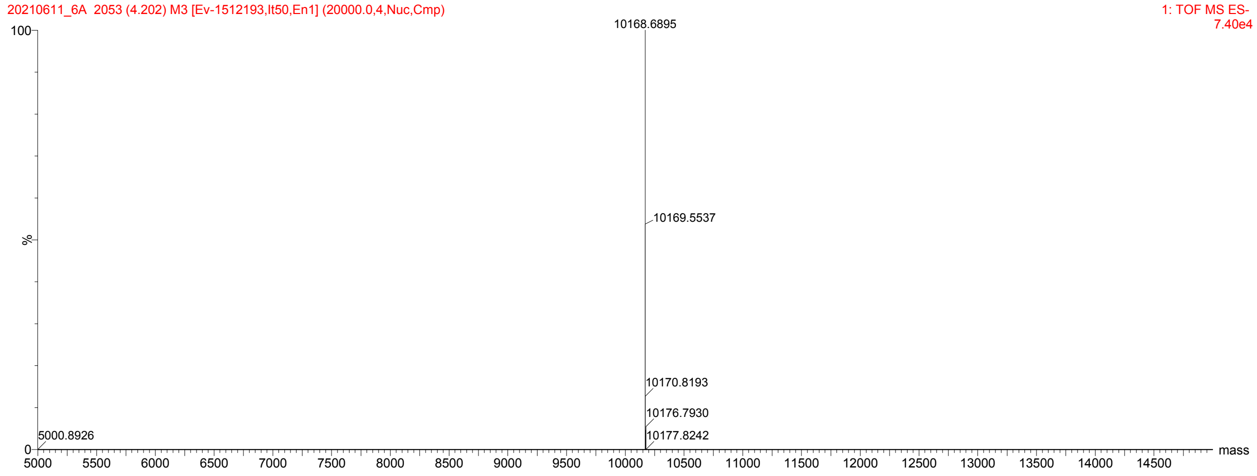

Figure S26. HR-MS of 3′-Cy5-5′-CRO-SMCC-DM1 conjugate.

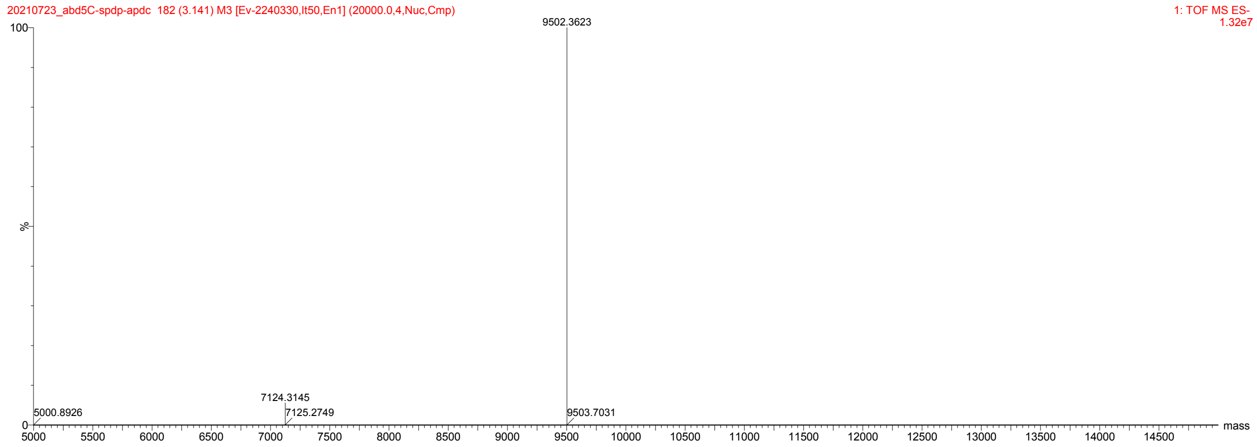

Figure S27. HR-MS of 5′-CRO-SPDP-DM1 conjugate.

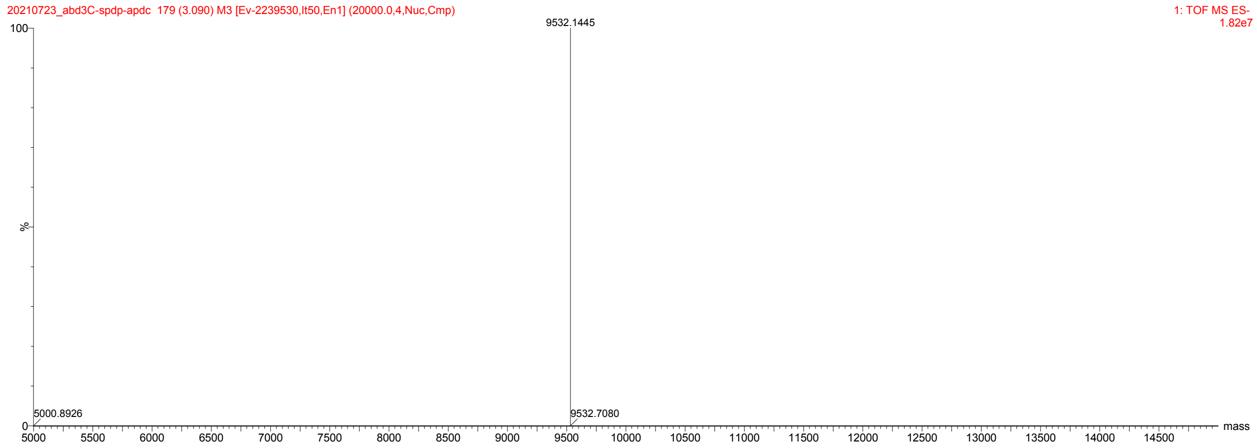

Figure S28. HR-MS of 3′-CRO-SPDP-DM1 conjugate.

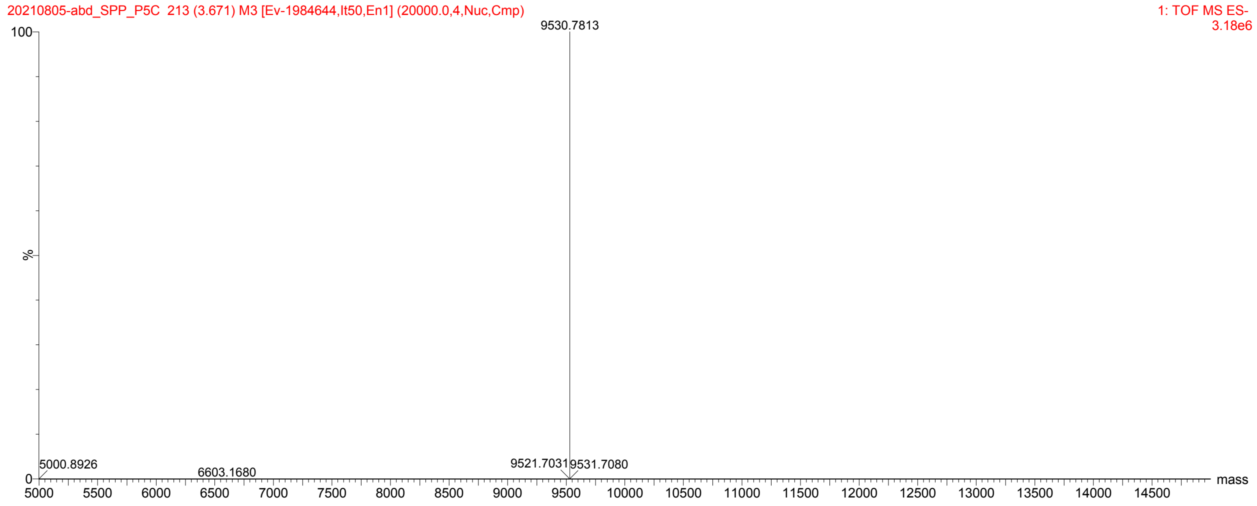

Figure S29. HR-MS of 5′-CRO-SPP-DM1 conjugate.

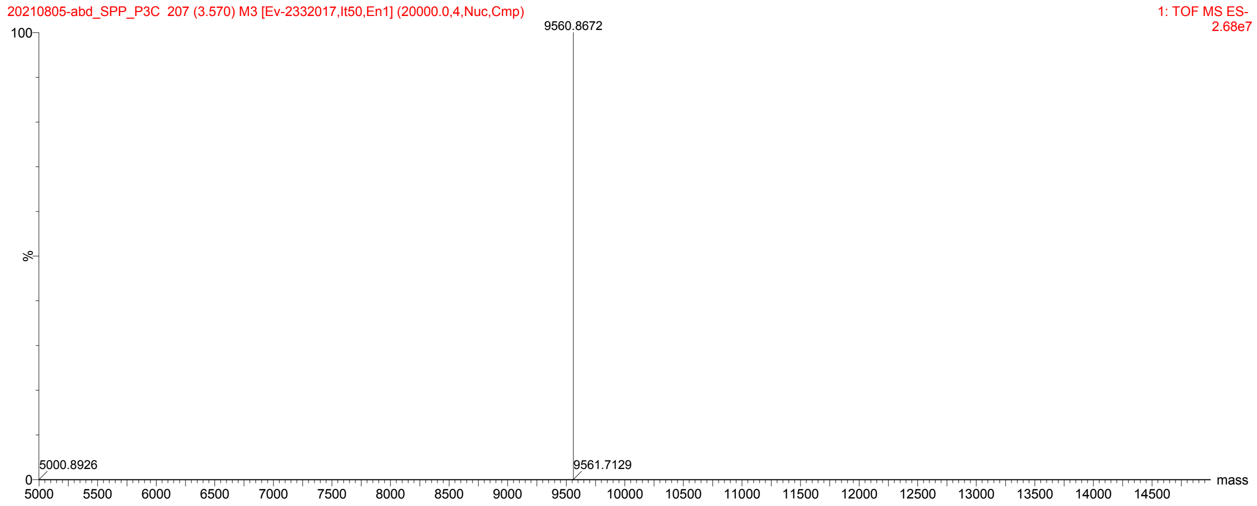

Figure S30. HR-MS of 3′-CRO-SPP-DM1 conjugate.

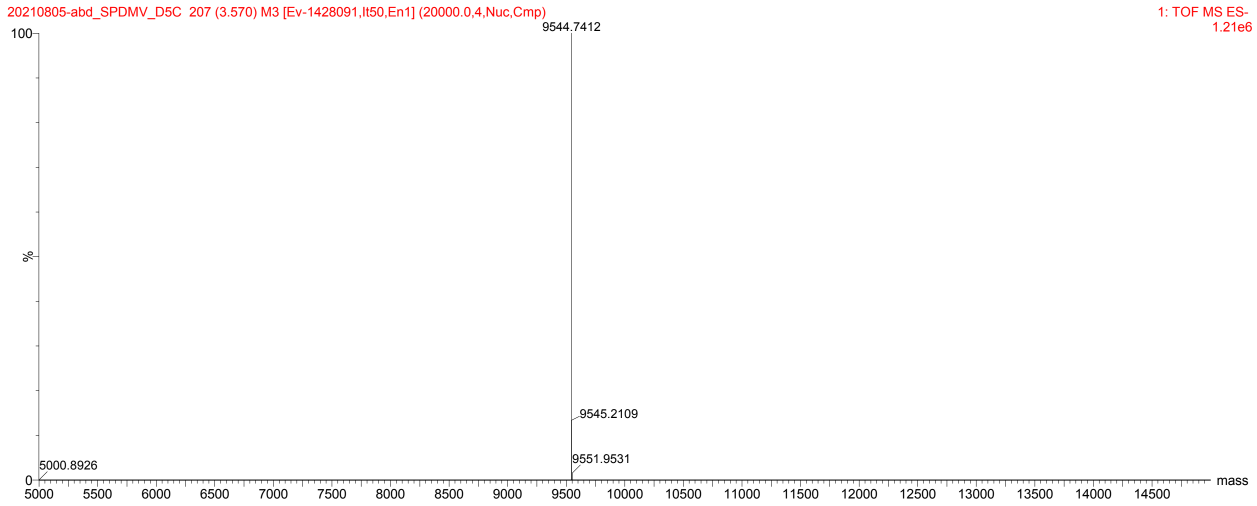

Figure S31. HR-MS of 5′-CRO-SPDMV-DM1 conjugate.

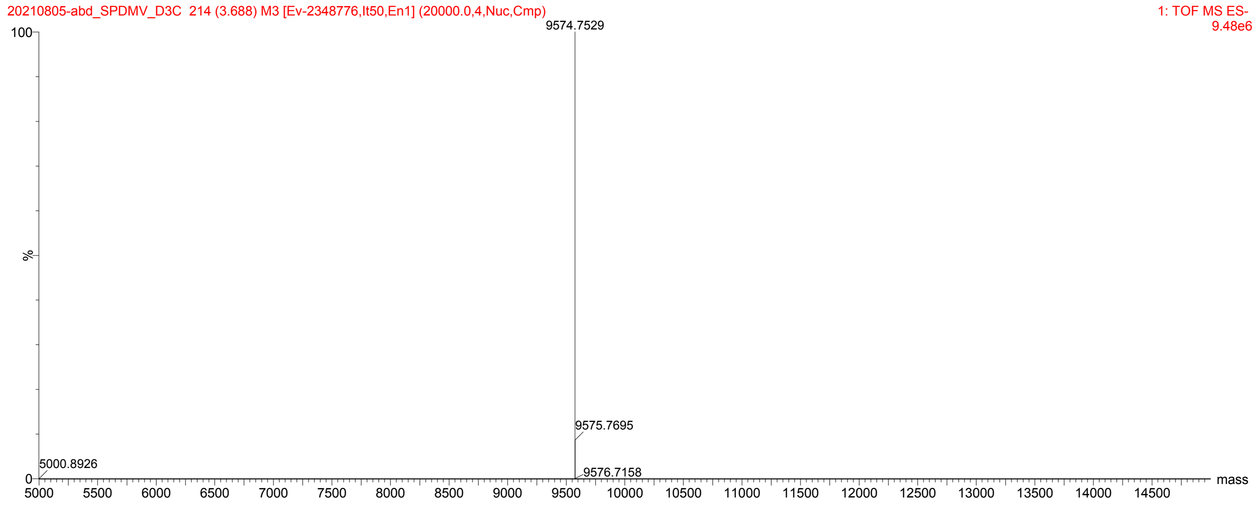

Figure S32. HR-MS of 3′-CRO-SPDMV-DM1 conjugate.

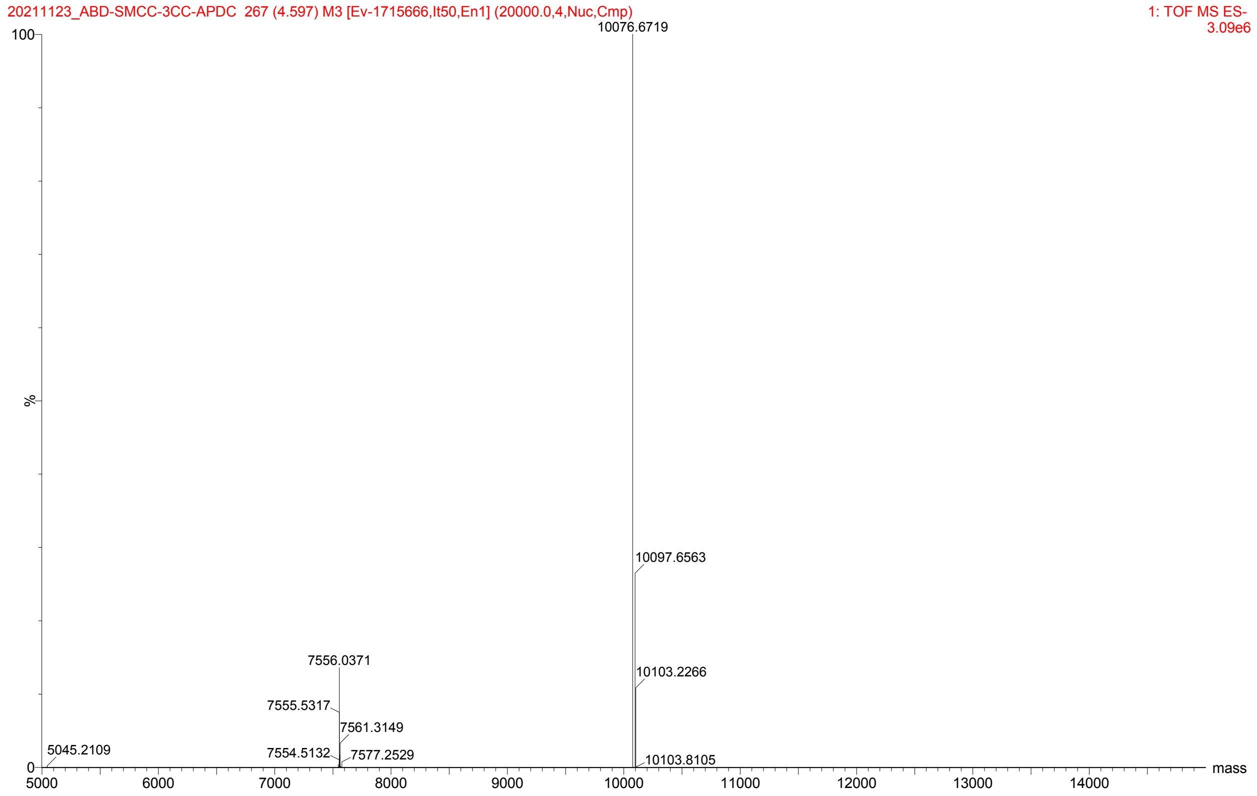

Figure S33. HR-MS of 3′-Cy5-5′-CRO-SPDMV-DM1 conjugate.
